# Identification and Analysis of *GhEXO* Gene Family Indicated That *GhEXO7_At* Promotes Plant Growth and Development Through Brassinosteroid Signaling in Cotton (*Gossypium hirsutum* L.)

**DOI:** 10.3389/fpls.2021.719889

**Published:** 2021-09-16

**Authors:** Shengdong Li, Zhao Liu, Guoquan Chen, Ghulam Qanmber, Lili Lu, Jiaxin Zhang, Shuya Ma, Zuoren Yang, Fuguang Li

**Affiliations:** ^1^Zhengzhou Research Base, State Key Laboratory of Cotton Biology, Zhengzhou University, Zhengzhou, China; ^2^State Key Laboratory of Cotton Biology, Institute of Cotton Research, Chinese Academy of Agricultural Sciences, Anyang, China; ^3^Saint John Paul the Great Catholic High School, Dumfries, VA, United States

**Keywords:** brassinosteroid, *GhEXO*, *G. hirsutum*, phylogenetic analysis, gene duplication, ectopic expression

## Abstract

Brassinosteroids (BRs), an efficient plant endogenous hormone, significantly promotes plant nutrient growth adapting to biological and abiotic adversities. BRs mainly promote plant cell elongation by regulating gene expression patterns. *EXORDIUM* (*EXO*) genes have been characterized as the indicators of BR response genes. Cotton, an ancient crop, is of great economic value and its fibers can be made into all kinds of fabrics. However, *EXO* gene family genes have not been full identified in cotton. 175 *EXO* genes were identified in nine plant species, of which 39 *GhEXO* genes in *Gossypium hirsutum* in our study. A phylogenetic analysis grouped all of the proteins encoded by the *EXO* genes into five major clades. Sequence identification of conserved amino acid residues among monocotyledonous and dicotyledonous species showed a high level of conservation across the N and C terminal regions. Only 25% the *GhEXO* genes contain introns besides conserved gene structure and protein motifs distribution. The 39 *GhEXO* genes were unevenly distributed on the 18 At and Dt sub-genome chromosomes. Most of the *GhEXO* genes were derived from gene duplication events, while only three genes showed evidence of tandem duplication. Homologous locus relationships showed that 15 *GhEXO* genes are located on collinear blocks and that all orthologous/paralogous gene pairs had *Ka* > *Ks* values, indicating purifying selection pressure. The *GhEXO* genes showed ubiquitous expression in all eight tested cotton tissues and following exposure to three phytohormones, IAA, GA, and BL. Furthermore, *GhEXO7_At* was mainly expressed in response to BL treatment, and was predominantly expressed in the fibers. *GhEXO7_At* was found to be a plasma membrane protein, and its ectopic expression in *Arabidopsis* mediated BR-regulated plant growth and development with altered expression of *DWF4*, *CPD*, *KCS1*, and *EXP5*. Additionally, the functions of *GhEXO7_At* were confirmed by virus-induced gene silencing (VIGS) in cotton. This study will provide important genetic resources for future cotton breeding programs.

## Introduction

Brassinosteroids (BRs), known as the sixth hormone, regulate a host of developmental process of plants including cell elongation and division and cell wall biosynthesis in plants (Yang et al., 2011; Saini et al., 2015). Provides a new way for crop improvement, the endogenous BR level has a significant impact on plant growth and development (Tanaka et al., 2005; Wei and Li, 2020). BR-deficient mutants show extreme dwarfing, delayed senescence, and decreased male fertility (Choe, 2006). BR mainly affects plant traits by promoting cell expansion and show a dose-specific effect in stimulating plant growth. Excessive application of bioactive BR will cause down-regulation of BR biosynthesis genes and up-regulation of BR inactivating genes, thereby hindering normal plant development (Tanaka et al., 2005).

Brassinosteroids in plant growth and development also play a crucial role by regulating gene expression patterns. cDNA and oligonucleotide microarrays have been used to identify BR-regulated genes (Müssig and Altmann, 2003). Some BR-response mediators (such as *BEE1*, *BEE2*, *BEE3*, and *BRH1*) are regulated at the transcription level by BR (Friedrichsen et al., 2002; Molnar et al., 2002). *EXORDIUM* (*AtEXO*) was initially identified as an indicator of BR response genes (Coll-Garcia et al., 2004). *EXO* was first found in *Arabidopsis* using a promoter trap insertional mutagenesis system (Farrar et al., 2003). *AtEM201* is a transgenic line with a T-DNA inserted into the *EXO* gene promoter. The relative transcription of *EXO* was significantly decreased in *AtEM201*, although the plant phenotype was normal (Farrar et al., 2003). The EXO-GUS fusion protein activity was high in apical meristems and young leaves (Farrar et al., 2003). Similarly, plants that specifically degraded *EXO* gene mRNA through RNA interference (RNAi) exhibited a normal phenotype (Coll-Garcia et al., 2004). However, *exo* knockout mutants exhibit a dwarf phenotype due to reduced cell expansion (Schroder et al., 2009). *EXO*-overexpression plants show enhanced vegetative growth and increased mRNA levels of BR-up-regulated genes, which is similar to the growth phenotype of BR-treated plants (Coll-Garcia et al., 2004). BR-deficient mutants exhibit weak *EXO* expression, while BR treatment of plants resulted in increased *EXO* transcript levels (Coll-Garcia et al., 2004). A BR hypersensitive mutant, *bes1-D*, shows constitutive *EXO* expression (Yin et al., 2002).

*EXO* and *EXO*-*Like* (*EXO/EXL*) genes encode eight homologous proteins in *Arabidopsis* (Schroder et al., 2009). *EXO* genes are *phosphate*-*induced*-*1*-*Like* (*PHI-1-Like*) genes, because the 300 amino acid conserved region of the EXO protein constitutes a phi-1 domain (PFAM entry PF04674) (Quan et al., 2019). The *PHI-1* genes were originally reported to be associated with tobacco cell cultures (Sano et al., 1999). Many plants contain *PHI-1* gene family members; there are 19 *PHI-1* genes in potato (*Solanum tuberosum* L.), 12 in rice (*Oryza sativa* L.), and 19 in maize (*Zea mays* L.) (Quan et al., 2019). *erg1* is a potato *PHI-1-Like* gene involved in ethylene and salicylic acid pathways (Dellagi et al., 2000). Overexpression of *EgPHI-1* significantly increased tolerance to NaCl, PEG, and mannitol treatments (Sousa et al., 2014). At present, there are very few papers reporting studies on *EXO* genes, and the functions of EXO proteins are unclear, particularly in cotton.

Cotton (*Gossypium* spp.) has gained immense attention for its natural fiber, and is the basis of a worldwide textile industry. In *Arabidopsis*, *EXO* family genes have been identified, but *EXO* genes have not been comprehensive and systematical analysis in cotton. With the advancement of genome sequencing technology, identified many different gene families in cotton become possible (Du et al., 2018; Ma et al., 2018; Yang et al., 2020).

In this study, the *EXO* gene family in four cotton species; *Gossypium hirsutum*, *Gossypium arboreum*, *Gossypium raimondii*, and *Gossypium barbadense* has been comprehensively and systematically analyzed. We then performed a phylogenetic analysis, and analyzed the sequences for conserved amino acids, gene structure, conserved protein motifs, chromosomal location, and genetic synteny. Furthermore, we preliminarily investigated tissue-specific expression profiles of *GhEXO* family members and examined their reacts to phytohormone stimuli. We then overexpressed *GhEXO7_At* in *Arabidopsis*, the subcellular localization of GhEXO7 has been determined in leaf epidermal cells of *Nicotiana benthamiana*, and used virus-induced gene silencing (VIGS) in cotton to explore the functions of the *GhEXO7_At* gene. The results of our study may help to elucidate the functions of the *GhEXO* genes in the growth and development of cotton.

## Materials and Methods

### *EXO* Gene Family Protein Sequence Identification

First, we downloaded the *Arabidopsis* EXO protein sequences from the TAIR database^1^ and used them as search queries to identify EXO protein-encoding genes in the genomes of different plant species including *G. hirsutum* (ZM24_ICR), *G. arboreum* (ICR, version 1.0), *G. raimondii* (JGI, version 2.0), *G. barbadense* (HAU, version 1.0), *Sorghum bicolor* (version 2.1), *Selaginella moellendorffii* (version 1.0), *Physcomitrella patens* (version 3.3), and *O. sativa* (version 7.0). The latest genome data for all species except *Arabidopsis* and cotton has been obtained from Phytozome (version 11)^2^. The *G. arboreum*, *G. hirsutum*, and *G. barbadense* genomic data were downloaded from the *Gossypium* Resource and Network Database^3^. We downloaded *G. raimondii* databases from the Cotton Functional Genomics Database^4^. The *Arabidopsis* database was downloaded from TAIR 10^5^. Next, all presumptive EXO protein sequences were verified by Interproscan 63.0^6^ (Jones et al., 2014) and SMART^7^ (Letunic et al., 2015). We then used ExPASy ProtParam^8^ to estimate the physicochemical properties of GhEXO family members.

### Conserved Sequence Logos and Phylogenetic Tree Construction

The WEBLOG was used for conservative amino acid residue analysis (Crooks et al., 2004). We use the maximum likelihood (ML) method which configured with default parameters by poisson model with MEGA X to construct the evolutionary tree (Kumar et al., 2012). We then used Evolview^9^ to visualize the phylogenetic tree (Subramanian et al., 2019).

### Gene Structure and Protein Conserved Motif Analysis

To determine the types of protein motifs, present and to perform gene structure analysis, the predicted GhEXO protein sequences were aligned with ClustalW, and a phylogenetic tree was produced with the NJ method as implemented in MEGA X. We analyzed *GhEXO* gene intron/exon structures using the Gene Structure Display Server 2.0 (Hu et al., 2015). In addition, using MEME online tool to forecast GhEXO protein domains (Bailey et al., 2006). TBtools^10^ software was used to visualize the distribution pattern of the conserved protein motifs (Chen et al., 2018).

### Chromosomal Location, Synteny, and *Ka/Ks* Analysis

First, we obtained the chromosomal position information for the *GhEXO* genes from the gff3 file of the cotton genome annotation data, and the genes were located on the chromosomes by MapInspect (He et al., 2012). We then used the GhEXO protein sequences seek queries against the whole genomes. The results were analyzed by MCScanX to generate collinearity pairs, which were visualized using Circos (version 0.69) software with the default parameters (Krzywinski et al., 2009). Finally, we use the PAML package to calculate the replacement rates of synonymous (Ks) and non-synonymous (Ka) (Yang, 2007).

### Cotton Tissue Expression Profile and Phytohormone Induction Treatment Assay

In our study, for the sake of confirm the expression pattern, we selected eight different tissues of *G. hirsutum* breed CRI24. Before phytohormone treatment, cotton seedlings at the cotyledon stage were transferred to a liquid medium and continued to grow the three-four leaf stage (Yang et al., 2014). Then we added the final concentration of 100 micromoles per liter of IAA, 100 micromoles per liter of GA, 10 micromoles per liter of BL to treat cotton seedlings for 1, 3, and 5 h. An equal volume of the corresponding solvent was added to the deionized water as a blank control.

Toteal RNA extraction used th *Trelifef*^TM^ RNAprep Pure Plant Plus Kit (Tsingke, Beijing, China). Reverse transcription of 1000 ng RNA is performed with the *EasyScript*^®^ All-in-One First-Strand cDNA Synthesis SuperMix (TransGen, Beijing, China) following the operating manuals. We used *GhHistone3* (GenBank accession no. AF024716) and *AtActin2* (AT3G18780.1) as internal controls for gene expression normalization. AceQ qPCR SYBR Green Master Mix (Low ROX Premixed) (Vazyme, Nanjing, China) was used for efficient amplification as a qRT-PCR on a Light-Cycler 480 (Roche Diagnostics, Germany). The data processing was used 2^–ΔΔCt^ method (Livak and Schmittgen, 2001). Details regarding all of the primers used in this study are provided in Supporting Information (Supplementary Table 1).

### Vector Construction, Subcellular Localization, and Creation of Transgenic Lines

To examine the function of *GhEXO7_At*, *Arabidopsis* (Col-0) plants were used to creation transgenic overexpressed *GhEXO7_At* lines. We amplified the full-length coding sequence (CDS) of *GhEXO7_At* gene from cDNA template. The full-length CDS was cloned into pCAMBIA-2301 driven by the 35S promoter. Next, we used the flower dip method to transform wild-type (Col-0) plants with *Agrobacterium tumefaciens* GV3101 (Clough and Bent, 1998). The transgenic lines were screened on 1/2 MS medium containing kanamycin (0.05 mg/mL) in a plant incubator at 22°C with a photoperiod set to 16 h/8 h. For VIGS, a 300 bp unique region of the *GhEXO7_At* CDS was cloned into the pTRV2 before transferred into *A. tumefaciens* (strain GV3101). Infiltration into upland cotton CRI24 was carried out as mentioned in previous study (Gao et al., 2011). The selected tissue was immersed in FAA fixation solution before dehydrate, transparency, tissues embedding in paraffin, and slices. Then, the sections were stained with toluidine blue, observed with Leica M165FC epifluorescence stereomicroscope (Leica Instruments GmbH).

To confirm the expression position of the GhEXO7_At-GFP fusion protein in cells, we cloned the full-length *GhEXO7_At* CDS into a vector to produce an in-frame GFP protein fusion. *N. benthamiana* leaves were co-infiltrated with the Agrobacterium strains together with the tombusvirus p19 suppressor of silencing (Witte et al., 2004). We left the plants in the dark for 24 h after co-infiltration. At 72 h after infiltration, stained with fluorescent membrane marker MM 4-64 [N-(3-Triethylammoniumpropyl)-4-(6-(4-(diethylamino) phenyl)hexatrienyl)pyridiniumdibromide] (AAT Bioquest, United States) for 8 min, GhEXO7_At was co-localized with MM 4-64 in *N. benthamiana* epidermal cells under fluorescence microscopy (Vida and Emr, 1995; Chen et al., 2015). *Arabidopsis* mesophyll protoplasts were isolated and transfected using the *Arabidopsis* Protoplast Preparation and Transformation Kit (Coolaber, Beijing, China) as described previously (Yoo et al., 2007; Ren et al., 2021). After 6–18 h of culture, *Arabidopsis* mesophyll protoplasts were observed with a fluorescence microscope.

## Results

### Identification of *EXO* Gene Family Members

We confirmed 21 nucleotide sequences in *G. arboreum*, 39 in *G. hirsutum*, 19 in *G. raimondii*, and 40 in *G. barbadense* as EXO genes. Additionally, we also identified the *EXO* genes in another dicotyledonous plant species (eight in *Arabidopsis*), two monocotyledonous species (12 in *O. sativa* and 12 in *S. bicolor*), moss (17 in *P. patens*), and seven in the lycophyte *S. moellendorffii*. We were surprised to discover that the number of *EXO* family members in diploid cotton (*G. arboretum* and *G. raimondii*) have almost half as tetraploid cotton (*G. hirsutum* and *G. barbadense*). In our study, we focused on the *EXO* genes in *G. hirsutum*. We first collected basic information on the *GhEXO* genes including start and end points, gene ID, chromosomal location of genes, the number of nucleotides in CDS, quantities of amino acid, and predicted molecular weight (MW) for the encoded proteins, isoelectric point (pI) is the pH of a protein solution when its net charge is zero, and Grand average of hydropathicity (GRVAY) values indicates the hydrophilicity and hydrophobicity of the protein (Table 1). The lengths of the predicted EXO proteins ranged from 246 (*GhEXO17_At*) to 384 (*GhEXO5_At*) amino acids, with MWs ranging from 27,297.26 Da (*GhEXO17_At*) to 41,826.73 Da (*GhEXO5_At*). The calculated pI values varied from 5.50 (*GhEXO13_At*) to 9.51 (*GhEXO19_Dt*). In addition, the minimum (−0.324) and maximum (0.091) GRAVY values were discovered for the *GhEXO17_At* protein and *GhEXO15_At* protein, respectively.

**TABLE 1:** Biophysical properties of the predicted GhEXO proteins.

Gene	Chromosome	Start	End	Gene ID	Amino acids	MW	pI	CDS (bp)	GRAVY
GhEXO1_At	A02	16182717	16183984	Ghicr24_A02G091400.1	317	34148.09	9.11	954	−0.092
GhEXO2_At	A02	16186238	16186238	Ghicr24_A02G091500.1	251	26778.33	7.62	756	0.066
GhEXO3_At	A02	98654637	98656045	Ghicr24_A02G172200.1	314	34000.48	9.25	945	−0.228
GhEXO4_At	A02	98658715	98660040	Ghicr24_A02G172300.1	317	34076.40	9.05	954	−0.225
GhEXO5_At	A03	100275420	100276887	Ghicr24_A03G197100.1	384	41826.73	6.81	1155	−0.019
GhEXO6_At	A04	76128643	76129987	Ghicr24_A04G132600.1	346	37444.55	6.19	1041	−0.038
GhEXO6a_At	A04	76148466	76149506	Ghicr24_A04G132800.1	346	37444.55	6.19	1041	−0.038
GhEXO7_At	A14	76558572	76559982	Ghicr24_A04G135700.1	296	32162.23	9.33	891	−0.024
GhEXO8_At	A05	14165826	14167071	Ghicr24_A05G150600.1	332	36098.95	6.50	999	−0.057
GhEXO9_At	A05	22626521	22627411	Ghicr24_A05G232900.1	296	31707.54	9.35	891	0.034
GhEXO10_At	A05	22647543	22648493	Ghicr24_A05G233000.1	316	33585.06	7.58	951	−0.034
GhEXO11_At	A05	22650823	22651782	Ghicr24_A05G233100.1	319	34215.10	9.24	960	−0.082
GhEXO11_At	A05	22650823	22651782	Ghicr24_A05G233100.1	319	34215.10	9.24	960	−0.082
GhEXO12_At	A09	36782625	36783999	Ghicr24_A09G058800.1	338	37293.82	8.75	1017	−0.052
GhEXO13_At	A10	4476821	4477968	Ghicr24_A10G048100.1	331	36533.28	5.50	996	−0.033
GhEXO14_At	A11	735920	737096	Ghicr24_A11G007500.1	339	37378.87	8.88	1020	−0.111
GhEXO15_At	A11	12174864	12175826	Ghicr24_A11G122000.1	320	34252.65	9.46	963	0.091
GhEXO16_At	A11	112386529	112387817	Ghicr24_A11G350300.1	303	33327.79	9.32	912	−0.038
GhEXO17_At	A12	59663413	59664307	Ghicr24_A12G102000.1	246	27297.26	8.41	741	−0.324
GhEXO18_At	A12	101488482	101489924	Ghicr24_A12G287500.1	339	37416.93	8.59	1020	−0.040
GhEXO19_At	A13	15397613	15398506	Ghicr24_A13G075800.1	297	32009.15	9.38	894	0.085
GhEXO1_Dt	D02	14299662	14300897	Ghicr24_D02G090600.1	317	34064.02	9.11	954	−0.089
GhEXO2_Dt	D02	14316384	14317328	Ghicr24_D02G090700.1	314	33486.76	6.36	945	−0.059
GhEXO3_Dt	D03	8230259	8231499	Ghicr24_D03G052900.1	314	33940.44	9.15	945	−0.206
GhEXO4_Dt	D03	8236456	8237662	Ghicr24_D03G053000.1	316	33973.32	8.96	951	−0.236
GhEXO5_Dt	D02	63919863	63921294	Ghicr24_D02G212900.1	349	37994.24	7.64	1050	−0.077
GhEXO6_Dt	D04	49542747	49544336	Ghicr24_D04G170000.1	350	37828.00	6.29	1053	−0.049
GhEXO7_Dt	D04	49945856	49947277	Ghicr24_D04G173400.1	296	32087.03	9.00	891	−0.004
GhEXO8_Dt	D05	12388849	12390143	Ghicr24_D05G145100.1	332	36107.92	6.20	999	−0.086
GhEXO9_Dt	D05	20052898	20053794	Ghicr24_D05G224000.1	270	28910.03	9.18	813	0.017
GhEXO10_Dt	D05	20068434	20069384	Ghicr24_D05G224100.1	316	33689.15	8.12	951	−0.099
GhEXO11_Dt	D05	20071545	20072504	Ghicr24_D05G224200.1	319	34151.95	9.08	960	−0.100
GhEXO12_Dt	D09	20573600	20575056	Ghicr24_D09G052500.1	338	37399.99	8.88	1017	−0.034
GhEXO13_Dt	D10	4303324	4304319	Ghicr24_D10G046100.1	331	36450.15	5.61	996	−0.045
GhEXO14_Dt	D11	666331	667688	Ghicr24_D11G007200.1	383	42426.83	9.16	1152	−0.090
GhEXO15_Dt	D11	10699616	10700569	Ghicr24_D11G117900.1	317	33956.25	9.51	954	0.056
GhEXO16_Dt	D11	65389650	65391024	Ghicr24_D11G335300.1	314	34271.82	9.00	945	0.007
GhEXO17_Dt	D12	14932789	14934053	Ghicr24_D12G072800.1	321	35249.56	6.25	966	−0.096
GhEXO18_Dt	D12	59435065	59436601	Ghicr24_D12G278500.1	339	37443.99	8.59	1020	−0.050
GhEXO19_Dt	D13	6672665	6673564	Ghicr24_D13G052800.1	299	32092.19	9.51	900	0.044

*MW: Molecular weight (in Da), pI: Isoelectric point. Biophysical properties of cotton EXO genes.*

### Phylogenetic Analysis of Proteins Encoded by the *EXO* Family Genes

**FIGURE 1:**
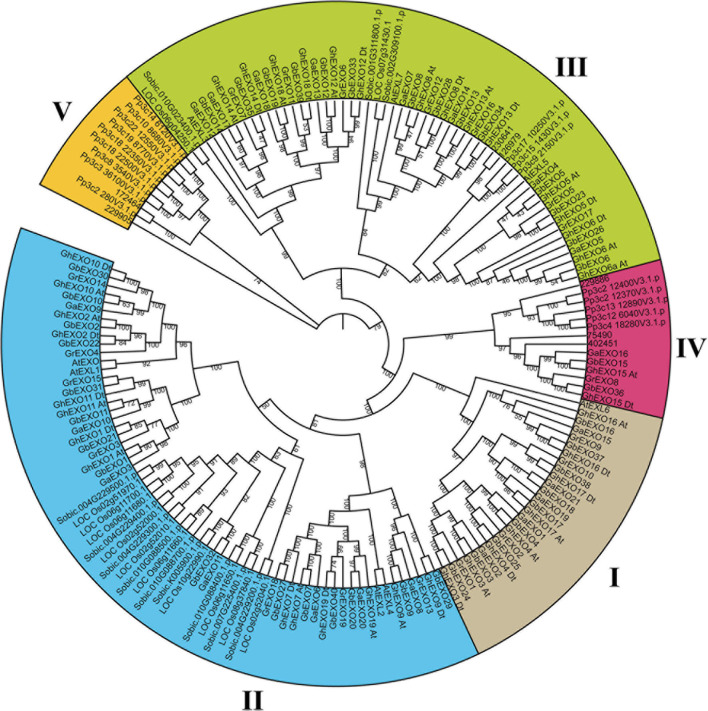
Phylogenetic analysis of predicted proteins encoded by the *EXO* genes from nine plant species. Phylogenetic analysis grouped all 175 EXO proteins into five clades in a tree calculated using the ML (maximum likelihood) method. Clades I–IV are shown in different colors. The prefixes At, Ga, Gh, Gr, Os, Zm, Sobic, Sm, and Pp are used to identify EXO family proteins from *A. thaliana*, *G. arboreum*, *G. hirsutum*, *G. raimondii*, *O. sativa*, *Z. mays*, *S. bicolor*, *S. moellendorffii*, and *P. patens*, respectively. In addition, At and Dt refer to the A and D sub-genomes of upland cotton (*G. hirsutum*), respectively.

In order to clarify the evolution of *EXO* genes among various species, an evolutionary tree containing nine representative species was constructed. The tree showed that all 175 predicted EXO proteins from the nine different plant species could be grouped into five major clades (Clades I-V) (Figure 1). The results of 175 EXO proteins analysis indicated that 8 of them were from *Arabidopsis thaliana* (*At*), 21 from *G. arboretum* (*Ga*), 39 from *G. hirsutum* (*Gh*), 19 from *G. raimondii* (*Gr*), 40 from *G. barbadense* (*Gb*), 12 from *O. sativa* (*Os*), 12 from *S. bicolor* (*Sobic*), 7 from *S. moellendorffii* (*Sm*), and 17 from *P. patens* (*Pp*).

In our analysis, Clade II included the most EXO proteins (66) followed by Clade III (57), Clade I (27), Clade IV (14), and Clade V (11). Although Clade II contained the most EXO proteins, with most of them coming from seven different species, the Clade III members come from all nine species included in the analysis. Most importantly, Clade I and Clade II contain only proteins from dicots and monocots, with no EXO proteins from either *S. moellendorffii* or *P. patens*, suggesting that these two groups may be specific to angiosperms. On the contrary, all 11 proteins in Clade V originate from the moss *P. patens* (9) and from *S. moellendorffii* (2). Interestingly, no algae homologs were identified, implying that the evolution of the *EXO* gene was accompanied by the emergence of bryophytes, and that the *EXO* genes present in higher plants may be derived from moss. Alternatively, the *EXO* genes could have been lost from the algal lineage. Clade IV contains proteins from the four cotton species, *P. patens*, and *S. moellendorffii*, while no monocot EXO proteins were present. For the purpose of further study in the evolution of EXO proteins among the cotton, phylogenetic analysis was performed in four cotton species (*G. hirsutum*, *G. arboreum*, *G. raimondii*, and *G. barbadense*) using the NJ method (Supplementary Figure 1). The whole EXO proteins in this tree were grouped into four clades (Clades I–IV).

Although the EXO proteins from dicots and monocots clustered close together, *G. hirsutum* and *G. barbadense* experienced a significant increase in *EXO* gene number, because the number of EXO proteins from these two cotton species was almost twice that from all other species in the phylogenetic analysis. Furthermore, a host of cheek by jowl clustering homologous genes were occurred in every species. This suggests that gene replication event is the dominant reason for the increase of *EXO* gene in plants.

### Conserved Amino Acid Residue Analysis

**FIGURE 2:**
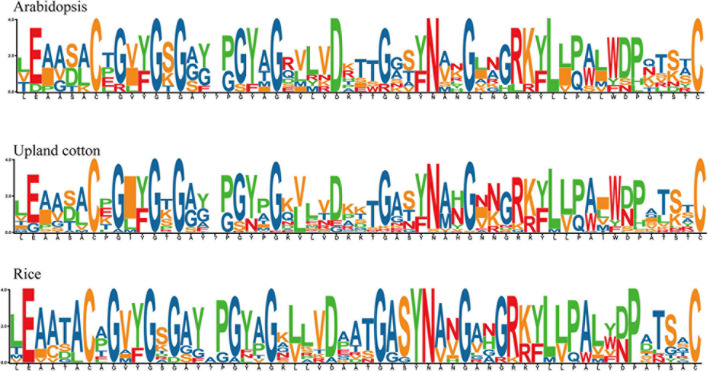
Sequence logo analysis of conserved amino acid residues in predicted EXO proteins. Sequence logos of conserved amino acid residues in the three plant species *Arabidopsis thaliana*, Upland cotton (*G. hirsutum*), and rice (*O. sativa*) showed highly conserved amino acids in EXO proteins during the evolution of dicot and monocot species.

In order to identify the homeodomain sequence and study the highly uniform amino acid sites in the GhEXO region, proteins from *At*, *Os*, and *Gh* were identified by multiple sequence alignments. The consequences showed that the distribution of amino acid residues in proteins from these three species was very similar at most positions. Specifically, it is highly conserved certain amino acid sites that covering E [2], C [7], G [9], G [12], G [14], G [22], D [27], G [31], Y [34], N [35], G [38], L [45], and C [57] were discovered. No specific conservative amino acid residue composition bias was observed in arbitrary position, indicating that in all observed species, no matter the N-terminal or C-terminal of the domain has an ultra-conservative distribution pattern (Figure 2).

### Chromosomal Distribution, Gene Duplication, and Gene Synteny Analysis

The position of the *GhEXO* gene on the cotton chromosomes can be predicted, through the analysis of the *GhEXO* genome sequence (GFF3 profiles). A total of 39 *GhEXO* genes were inhomogeneous distribution on 18 chromosomes, 20 and 19 of them distributed on the At and Dt sub-genome chromosomes, severally (Supplementary Figure 2). The most genes (four genes each) were located on homologous chromosomes A05 and D05. Seven chromosomes out of 18 contained only one *GhEXO* gene. Among the 39 *GhEXO* genes, only the orthologs of *GhEXO3*, *GhEXO4*, and *GhEXO5* are not located on the homologous sub-genome chromosomes of At and Dt. Previous study has shown that translocation events occur on chromosomes A02 and A03 explaining the fact of *GhEXO3_At*, *GhEXO4_At*, and *GhEXO5_At*, happen to be on the A02 and A03 chromosomes, are distributed on non-homologous chromosomes compared with its orthologs on the sub-genome chromosomes of Dt (Zhang et al., 2015).

**FIGURE 3:**
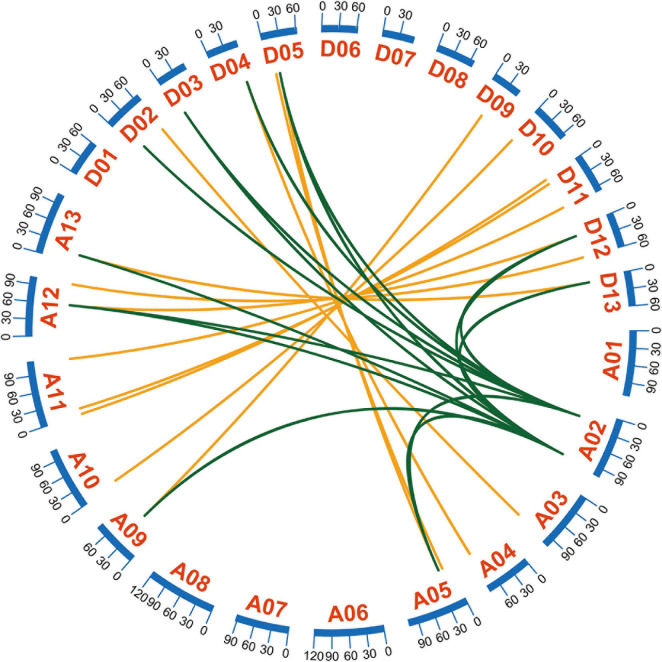
Collinearity analyses of *G. hirsutum EXO* genes. Collinearity analyses of the *GhEXO* genes on the 18 chromosomes of *G. hirsutum*. A01–A13 represent chromosomes of the At sub-genome and D01-D13 correspond to chromosomes of the Dt sub-genome. Gene pairs formed by segmental duplication within the At and Dt sub-genomes are linked by green lines. The orange lines connect gene pairs identified on homologous chromosome pairs from the At and Dt sub-genomes.

An MCSCAN analysis can be used to detect various types of gene duplication (Yang et al., 2018). Tandem duplication, segmental duplication, and whole-genome duplication contribute to gene family expansion (Xu et al., 2012). A typical allotetraploid derived from two diploid species, *G. hirsutum*, is an indispensable material for analyzing polyploid effects (Paterson et al., 2012). Most *GhEXO* genes were identified as resulting from whole genome duplication (WGD) or segmental duplication (Supplementary Table 2). Three genes might have undergone tandem duplication (Supplementary Table 2). To identify the homologous locus relationships on different sub-genomes, we proceeded a collinearity analysis. Our results indicate that 15 pairs of genes are present in colinear blocks (Figure 3). Among the duplicated genes, *GhEXO3_At* formed eight pairs of genes with significant collinearity and *GhEXO1_At* formed seven pairs of genes with significant collinearity, indicating that they were active during evolution (Figure 3). There were no singleton and proximal duplications detected in the chromosomal regions containing the *GhEXO* genes (Supplementary Table 2).

Cotton species have experienced genome expansion during evolution, and the number of genes continues to increase. Duplicated genes may have undergone functional differentiation, including partial or complete loss of the previous functions, gaining new functions or maintaining previous functions (Vandepoele et al., 2003). The non-synonymous (*Ka*) and synonymous substitution (*Ks*) ratios (*Ka/Ks*) of *GhEXO* genes allowed us to determine which kind of selection pressure (positive selection, negative selection, or purifying selection) acted on it. When *Ka* < *Ks*, the genes were selected for purification, indicating that there was no natural selection pressure; when *Ka* = *Ks*, the genes neutral evolution; and *Ka* > *Ks*, genes are strongly subject to positive selection show that rapid recent genetic evolution. The *Ka/Ks* ratios of 22 homologous pairs, including three paralogous pairs that arose from WGD or segmental duplication and 19 orthologous pairs were calculated. All the values were found to be <1 (Table 2), indicating that the *GhEXO* genes experienced purifying selection pressure, and that the protein functions may be conserved after gene family expansion.

**TABLE 2:** The Ka and Ks values for homologous gene pairs in cotton.

Paralogous	Ka	Ks	Ka/Ks
GhEXO3_At/GhEXO17_At	0.354	1.735	0.204
GhEXO1_At/GhEXO6_Dt	0.757	4.174	0.181
GhEXO3_At/GhEXO17_Dt	0.368	1.521	0.242
GhEXO1_At/GhEXO1_Dt	0.004	0.039	0.108
GhEXO2_At/GhEXO2_Dt	0.022	0.037	0.586
GhEXO3_At/GhEXO3_Dt	0.024	0.062	0.388
GhEXO4_At/GhEXO4_Dt	0.024	0.056	0.424
GhEXO5_At/GhEXO5_Dt	0.004	0.043	0.090
GhEXO6_At/GhEXO6_Dt	0.009	0.057	0.158
GhEXO7_At/GhEXO7_Dt	0.009	0.049	0.184
GhEXO8_At/GhEXO8_Dt	0.007	0.025	0.269
GhEXO9_At/GhEXO9_Dt	0.020	0.046	0.441
GhEXO10_At/GhEXO10_Dt	0.014	0.021	0.664
GhEXO11_At/GhEXO11_Dt	0.006	0.044	0.127
GhEXO12_At/GhEXO12_Dt	0.010	0.051	0.205
GhEXO13_At/GhEXO13_Dt	0.008	0.017	0.472
GhEXO14_At/GhEXO14_Dt	0.012	0.033	0.364
GhEXO15_At/GhEXO15_Dt	0.013	0.025	0.506
GhEXO16_At/GhEXO16_Dt	0.017	0.148	0.117
GhEXO17_At/GhEXO17_Dt	0.034	0.137	0.249
GhEXO18_At/GhEXO18_Dt	0.009	0.055	0.167
GhEXO19_At/GhEXO19_Dt	0.015	0.071	0.214

*Ka: non-synonymous, Ks: synonymous.*

### Exon/Intron Organization and Conserved Motif Analysis

According to previous studies, gene structure can reflect evolutionary relationships between different plant species and the exon/intron distribution patterns of genes is associated with their biological functions (Roy and Gilbert, 2006). To further explore the phylogenetic relationships and potential protein functions amongst the *GhEXO* genes, we performed exon/intron structure and conserved motif analyses (Supplementary Figure 3). Our results indicate that most of the *GhEXO* genes with similar exon/intron numbers and distribution patterns belong to the same subfamilies. Most of the *GhEXO* genes have no introns. Ten of the 39 *GhEXO* genes analyzed in this study contain introns, accounting for 25% of the total *GhEXO* genes (Supplementary Figure 3B). To investigate the conserved motif distribution pattern in the GhEXO proteins, we constructed a phylogenetic tree with the neighbor joining method to couple with the domain positions (Supplementary Figure 3A). The results showed that most GhEXO proteins have similar motif distribution patterns; for example, motifs 3, 4, and 7 were present in all proteins (Supplementary Figure 3A). Motif 8 was only detected in the 15 proteins of Clade III, while motif 10 was absent. Furthermore, motif 10 was present in all GhEXO proteins of Clades I and II except in GhEXO2_At/Dt and GhEXO10_At/Dt. The two members of Clade IV lacked both motif 5 and motif 8. In general, the motif distribution patterns of the same sub-family GhEXO proteins in phylogenetic trees were similar.

### Responses of *GhEXO* Members Under Phytohormonal Treatments and Its Expression Profile Analysis in Disparate Tissues

**FIGURE 4:**
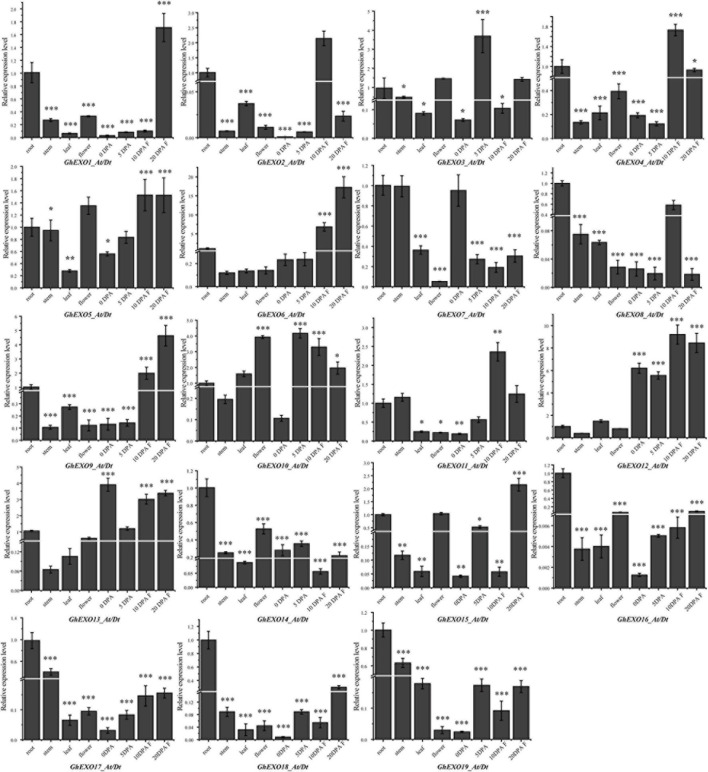
Tissue specific expression pattern analysis of *GhEXO* genes. Expression of 19 *GhEXO* genes in four different tissues and in fibers at 0, 5, 10, and 20 days after anthesis (DPA) as determined by qRT-PCR. Error bars represent the standard deviations of three independent experiments. Significant differences were determined by Student’s *t*-test: ^∗^*p* < 0.05, ^∗∗^*p* < 0.01, ^∗∗∗^*p* < 0.001.

The expression level of functional genes in various tissues is closely related to plant phenotype. Therefore, we investigated the expression profiles of *GhEXO* genes in eight different cotton tissues (root, stem, leaf, flower, and 0, 5, 10, and 20 DPA fiber) using qRT-PCR (Figure 4). Based on the expression patterns, *GhEXO12* (the gene on At and Dt sub-genomes) and *GhEXO13* were found to be preferentially expressed in all tested fiber tissues. However, *GhEXO10* was expressed at 5, 10, and 20 DPA (days post-anthesis), *GhEXO5* was expressed at 10 and 20 DPA, while *GhEXO1*, *GhEXO6*, *GhEXO9*, and *GhEXO15* were expressed only at 20 DPA. Similarly, *GhEXO2*, *GhEXO4*, *GhEXO11*, and *GhEXO12* were expressed only at 10 DPA. *GhEXO3* was mainly expressed at 5 DPA, while *GhEXO7* was preferentially expressed in 0 DPA fiber tissue. *GhEXO8*, *GhEXO14*, *GhEXO16*, *GhEXO17*, *GhEXO18*, and *GhEXO19* were advantageous expressed in the roots.

**FIGURE 5:**
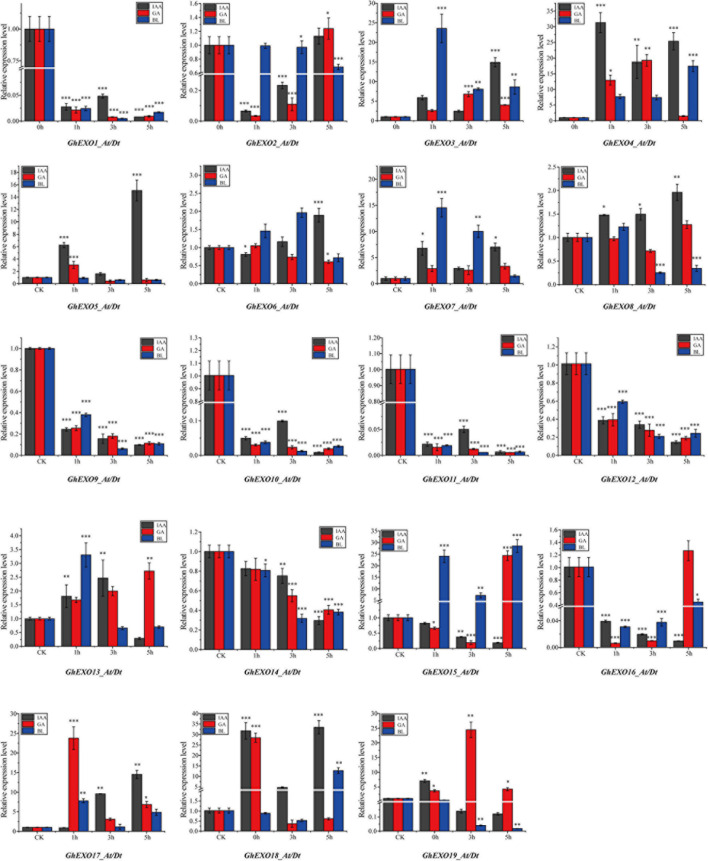
Expression pattern analysis of *GhEXO* genes under hormone treatment. Expression patterns of 19 *GhEXO* genes in response to three hormone treatments (IAA, GA, and BL) as determined by qRT-PCR analysis. The error bars show the standard deviations of three independent repeats. Student’s *t*-test: ^∗^*p* < 0.05, ^∗∗^*p* < 0.01, ^∗∗∗^*p* < 0.001.

Furthermore, exposure to different phytohormones (Auxin: IAA, Gibberellins: GA3, and Brassinosteroids: BL) revealed variable expression patterns in *GhEXO* family members. As shown in Figure 5, *GhEXO3*, *GhEXO4*, *GhEXO7*, and *GhEXO17* were upregulated while *GhEXO1*, *GhEXO9*, *GhEXO10*, *GhEXO11*, *GhEXO12*, and *GhEXO14* were completely downregulated in response to all tested hormones at all sampled time points. However, *GhEXO5* and *GhEXO18* showed up-regulation at all points after IAA treatment. *GhEXO19* was significantly induced by GA, especially at the 3 h time point. Interestingly, *GhEXO7* and *GhEXO15* responded significantly to BL, especially at the 1 h time point. *GhEXO7* was predominantly expressed at 0 DPA. Moreover, *GhEXO7*, gene on At and Dt sub-genomes, and *AtEXO* are extreme resemblance and may play similar functions in plants. Therefore, we selected the *GhEXO7* gene for further functional characterization.

### Subcellular Localization of the *GhEXO7_At*, Ectopic Expression, and VIGS of *GhEXO*s

**FIGURE 6:**
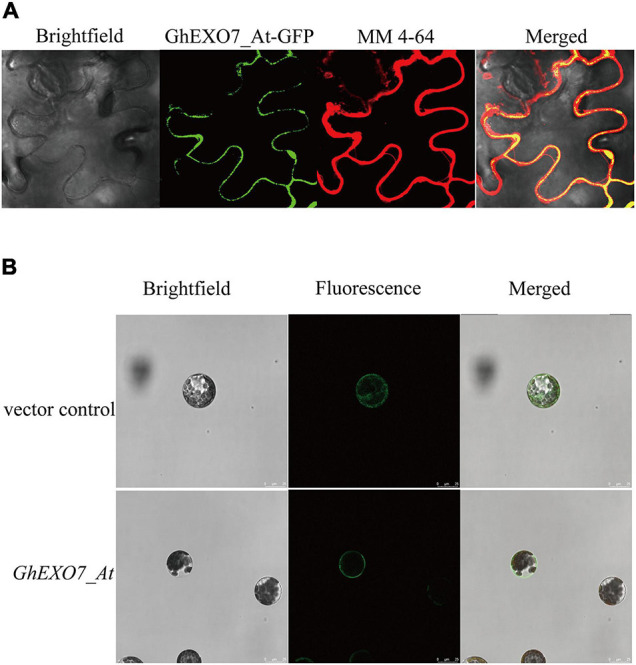
Subcellular localization of GhEXO7_At. **(A)** The Brightfield, GhEXO7_At-GFP Fluorescence, MM 4-64, and Merged images of infiltrated *N. benthamiana* leaf epidermal cells are shown. **(B)** The empty vector (control) and the GhEXO7_At-GFP vector were introduced into *Arabidopsis* mesophyll protoplasts. The Brightfield, Fluorescence (GFP), and Merged images are shown.

Above all, we determined the localization of the GhEXO7_At protein in *N. benthamiana* leaf epidermal cells and in *Arabidopsis* mesophyll protoplasts in order to further investigate the roles of *GhEXO* gene family members. The epidermal cells of *N. benthamiana* leaves were stained with an amphiphilic small molecule styrene dye MM 4-64 (Nguyen et al., 2018; Lichius and Zeilinger, 2019). The results of confocal laser scanning microscopy showed that the green fluorescence excited by EXO7_At-GFP protein was basically consistent with the red fluorescence of MM 4-64, membrane marker, indicated that GhEXO7_At is localized to the plasma membrane as compared to the MM 4-64 (Figure 6A). We also found that GhEXO7_At is localized to the cell membranes in *Arabidopsis* protoplasts (Figure 6B). From these results, we conclude that GhEXO7_At is a plasma membrane-localized protein.

**FIGURE 7:**
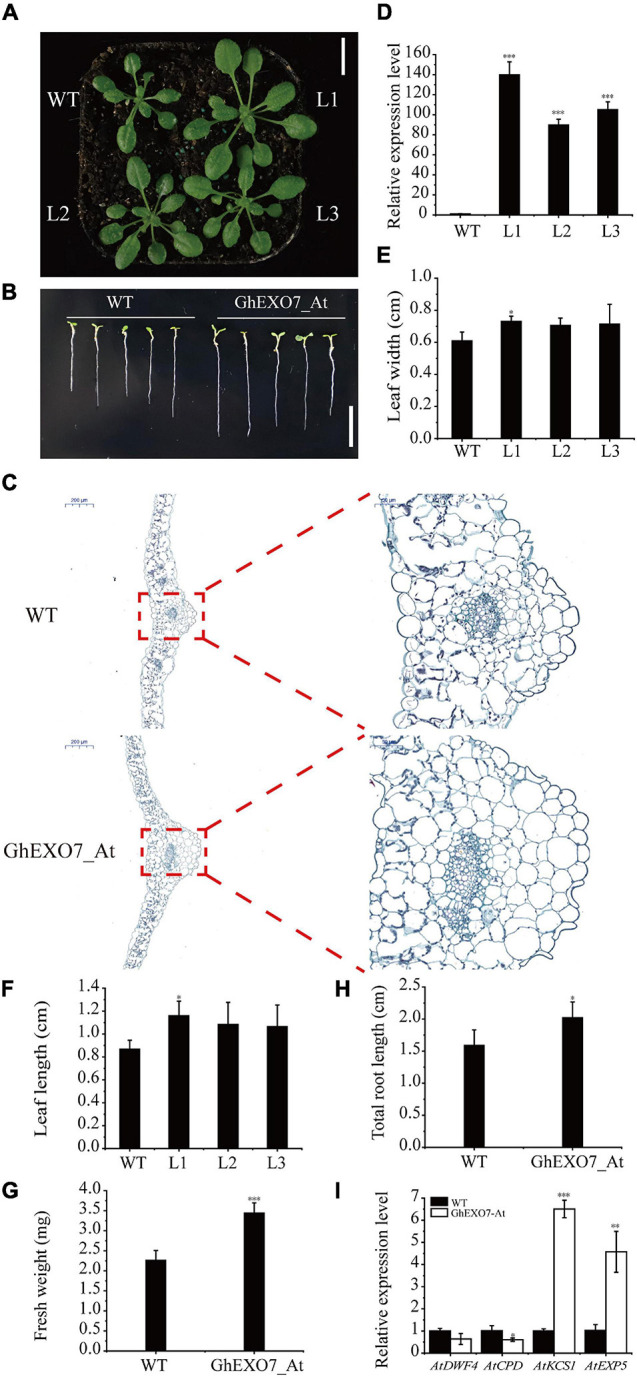
Phenotypes of plants from *Arabidopsis* lines overexpressing *GhEXO7_At*. **(A)** Phenotypic comparison of three *GhEXO7_At* lines and Col-0 (WT). Scale bars = 1 cm. **(B)** The root phenotypes of WT and *GhEXO7_At*-OE plants grown 14 days in the 1/2 MS. Scale bars = 1 cm. **(C)** Comparison of cells size from the fifth leaf of *GhEXO7_At*-OE and WT. **(D)** Relative *GhEXO7_At* expression levels in Col-0 and *GhEXO7_At* transgenic plants. Width **(E)** and length **(F)** of the fifth rosette leaf in WT and *GhEXO7_At* overexpression plants, Student’s *t*-test: ^∗^*p* < 0.05. Comparison of plant fresh weight **(G)** and total root length **(H)** of *GhEXO7_At*-OE and WT. **(I)** Relative expression levels of BR-repressed genes (*CPD* and *DWF4*) and BR-induced genes (*EXP5* and *KCS1*) in Col-0 and the *GhEXO7_At* transgenic plants. Significant differences in expression were determined by Student’s *t*-test: ^∗^*p* < 0.05, ^∗∗^*p* < 0.01, ^∗∗∗^*p* < 0.001.

To confirm the role of the *GhEXO7_At* gene in plant growth and development, the *GhEXO7_At* CDS was transformed in *Arabidopsis* Col-0 plants under the control of the CaMV 35S promoter. The transgenic lines over expressing *GhEXO7_At* showed enhanced growth compared to wild-type (WT) plants (Figure 7A). We also determined the relative expression level of *GhEXO7_At* estimate the relative degree of overexpression. The qRT-PCR results confirmed that the *GhEXO7_At* gene expression level was many folds higher in the overexpression lines compared to the WT control (Figure 7D). The lengths and widths of the fifth rosette leaves were increased in the *GhEXO7_At*-overexpression lines compared to the control (Figures 7E,F). Leaf lengths in lines L1, L2, and L3 were 34, 25, and 23% longer and were 20, 16, and 17% wider compared to WT, respectively. In addition, according to the statistics, its roots length was 27% longer on average than WT, and the fresh weight was significantly increased by 52.2% when it was grown on MS medium for 14 days (Figures 7B,G,H). The transgenic lines showed enhanced growth was also verified in the leaf cells histological observations experiment (Figure 7C). According to report, BR promotes plant growth and cell elongation; therefore, we performed expression analyses of BR biosynthesis genes (*DWF4* and *CPD*), the fatty acid elongase gene *KCS1*, and the cell elongation gene *EXP5* in the transgenic *Arabidopsis* lines. The results showed that *DWF4* and *CPD* expression was downregulated while the expression of *KCS1* and *EXP5* was upregulated in the transgenic *GhEXO7_At*-overexpressing Arabidopsis lines (Figure 7I).

**FIGURE 8:**
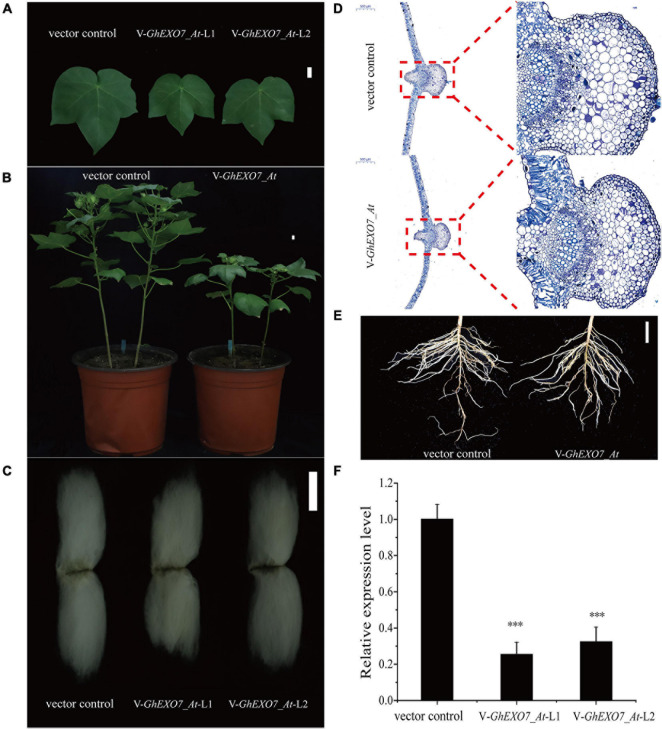
Virus-induced gene silencing (VIGS) of *GhEXO7_At* in cotton. **(A)** Phenotypes of leaves of the vector control and VIGS: *GhEXO7_At* plants. Scale bars = 1 cm. **(B)** Plant architecture of the vector control and VIGS: *GhEXO7_At* plants. Scale bars = 1 cm. **(C)** Fibers produced by the vector control and VIGS: *GhEXO7_At* plants. Scale bar = 1 cm. **(D)** Comparison of cells size from the fourth leaf of vector control and VIGS: *GhEXO7_At* plants. **(E)** The root phenotypes of vector control and VIGS: *GhEXO7_At* plants grown 30 days in the greenhouse. **(F)** Relative expression levels of the *GhEXO7_At* gene in the vector control and VIGS: *GhEXO7_At* plants. Student’s *t*-test: ^∗∗∗^*p* < 0.001.

To further explore the function of *GhEXO7_At*, a VIGS vector carrying a region of the *GhEXO7_At* gene and the empty vector control were individually inoculated into cotton seedlings at the 2-leaf stage. The *GhEXO7_At* gene was silenced, which was verified by quantifying the relative expression levels in the infected cotton plants. We found that *GhEXO7_At* gene expression was decreased by 70–80% in the silenced plants (two randomly hosen lines VIGS-*GhEXO7_At*-1 and VIGS-*GhEXO7_At*-2) as compared to the control plants (Figure 8F). We also examined the expression of other *GhEXO* genes from the same phylogenetic clade as *GhEXO7_At*. The expression levels were reduced to varying degrees in the silenced plants (Supplementary Figure 4). The role of *GhEXO7_At* and its subfamily genes in plant growth and development was confirmed by results showing that leaf length, width (Figure 8A and Supplementary Figure 6A,B) decreased to varying degrees compared with vector control plants, leaf cells histological observations showed that the cells in the silenced plants were small and cell expansion was limited (Figure 8D). Furthermore, plant height (Figure 8B and Supplementary Figure 6C), fiber length (Figure 8C and Supplementary Figure 6D), root length, and root fresh weight (Figure 8E and Supplementary Figure 6E,F) were reduced compared to the VIGS vector control plants. The expression of *GhEXO7_At/Dt* was most significantly down-regulated, so *GhEXO7* serves the main function.

## Discussion

As an important economic crop, cotton (*G. hirsutum*) is cultivated around the globe, providing an important source of raw fiber for the textile industry. When grown in the field, cotton plants are exposed to a variety of biotic and abiotic stresses. In recent years, considerable progress has been made in cotton genomics research, which has resulted in reference genome sequences for many diploid (Wang et al., 2012; Li et al., 2014) and all allotetraploid cotton species (Paterson et al., 2012; Li et al., 2015; Zhang et al., 2015; Hu et al., 2019; Wang et al., 2019; Chen et al., 2020; Huang et al., 2020). In addition, re-sequencing studies have included the wide range of cotton verities, cultivars, and land races (Du et al., 2018; Ma et al., 2018). Many gene families in cotton, including *GhPERK* (Qanmber et al., 2019b), *GhGGPPS* (Ali et al., 2020), *GhHH3* (Qanmber et al., 2019a), *GhAAI* (Qanmber et al., 2019c), *GhBES1* (Liu et al., 2018), *GhSnRK2* (Liu et al., 2017), *GhRING-H2* (Qanmber et al., 2018), *GhIDD* (Ali et al., 2019), *MIKC-type MADS-Box* (Ren et al., 2017), *GhKLCR* (Li et al., 2019), *GhGSK* (Wang et al., 2018), and *GhGH3* (Yu et al., 2018), among others, have been studied. Compared with other plant hormones, BR has high physiological activity and low content in plants, and it strongly induces nutritional and reproductive growth (Qanmber et al., 2019c). *EXO* gene family members have been shown to perform diverse functions in *Arabidopsis* in addition to the regulation of BR responsive genes. The results of our study identified the *GhEXO* family in the upland cotton to provide the foundation for deeper investigations into the functions of *GhEXO* genes in cotton.

### *GhEXO* Gene Evolution

In the present study, for the first time, we identified the *EXO* gene family in cotton and analyzed its properties. It is believed that, around 5 to 10 Mya (million years ago), hybrids between diploid cotton of two different species gave rise to the heterotetraploid variety of cotton *G. hirsutum* and *G. barbadense*. *G. arboretum* evolved into the At subgenome and *G. raimondii* into the Dt gene subgroup (Li et al., 2015; Huang et al., 2020, 2021; Yang et al., 2020). In this study, total 175 *EXO* genes were identified in different varieties of cotton (*Ga*, *Gh*, *Gr*, and *Gb*), one other dicotyledonous species (*Arabidopsis*), two monocotyledonous species (*O. sativa* and *S. bicolor*), a moss (*P. patens*), and a lycophyte (*S. moellendorffii*). Interestingly, the number of *EXO* genes identified in the two allotetraploid cotton species *G. hirsutum* and *G. barbadense* was almost the sum of the number of *EXO* genes in diploid cotton varieties *G. arboreum* and *G. raimondii*, which strengthens the conclusion that the allotetraploid cotton species are derived from the hybridization and polyploidization of two diploid species (Yang et al., 2020; Huang et al., 2021). Next, the genome size of *G. arboreum* is larger than the genome of *G. raimondii* (Huang et al., 2021), and in this regard more *EXO* genes were identified in *G. arboreum* than in *G. raimondii*. Furthermore, the number of *EXO* gene family members were same in the two included monocot species (*O. sativa* and *S. bicolor*), and together with these findings we can speculate that the *EXO* gene family was conserved during evolution. Additionally, the presence of *EXO* genes in the two primitive plants *P. patens* and *S. moellendorffii* indicate that the *EXO* genes constitute an ancient gene family that has persisted during evolution in the plant kingdom. And with the substantial chromosome rearrangements in eukaryotes and cotton-specific whole-genome duplications, it has expanded and evolved in the cotton genome.

Our phylogenetic analysis of EXO protein sequences from the nine different plant species classified all EXO proteins into five major clades (I–V). Clade II contains the most EXO family members, while Clade V contains the least. More precisely, Clade III contains EXO proteins from all species included in the analysis (dicots, monocots, moss, and lycophyte), while Clades I and II contain only dicot and monocot EXO proteins, demonstrating that Clade III is the most primitive group, and that the proteins in Clades I and II may be specific to angiosperms. In contrast Clade V only contains EXO proteins from the primitive plants *P. patens* and *S. moellendorffii*. The absence of *EXO* genes in algae leads us to hypothesize that the *EXO* genes might have appeared in moss after the separation of moss and algae during evolution. Furthermore, the phylogenetic analysis of all the EXO proteins from the four cotton species divided them into four clades, corresponding to Clade I–IV except Clade V in the evolution analysis of different species. However, Clade III contains the most genes, although Clade II also contains numerous genes. These two clades contain almost 70% of the cotton *EXO* genes, which may be related to the fact that the *EXO* genes of each species are not evenly distributed in each clade. This phylogenetic analysis of cotton distinctly showing the clustering of orthologous/paralogous gene pairs and supporting the hybridization and polyploidization of cotton species (Li et al., 2015; Zhang et al., 2015; Huang et al., 2021).

Conserved amino acid residue analysis in the two dicot species *Arabidopsis* and *G. hirsutum* and the monocot species rice (*O. sativa*) revealed remarkably similar N- and C-terminal amino acid residues, demonstrating that the EXO proteins are highly conserved in dicots and monocots and the composition of each amino acid is not detected to be biased in any area. This corresponds to the last two motifs in the analysis of upland cotton conserved motifs, which are contained in all GhEXO protein sequences. Moreover, it has been found that the structure of a gene permits its evolutionary analysis, and the pattern of exon/intron distribution is useful for its function (Roy and Gilbert, 2006; Liu et al., 2021). In our study, exon/intron analysis indicated that most of the *GhEXO* genes have no introns, only 25% or fewer contain introns. Similarly, conserved protein motif distribution patterns were observed in almost all of the GhEXO proteins, and the proteins exhibiting similar gene structure and protein motif distribution pattern were generally in the same clade. Previous analyses of the cotton *WOX* (Yang et al., 2017) and *YABBY* gene families (Yang et al., 2018) showed similar results. Taken together, these findings strongly suggest that the *EXO* gene family has been conserved in plants during the course of evolution.

### Expansion of the *GhEXO* Gene Family

During plant evolution, many gene families such as *GRX* (Malik et al., 2020) and *AAI* (Qanmber et al., 2019c) underwent expansion, and the number of family members increased compared to related species or, in the case of polyploids, their parental species. Here, from the phylogenetic analysis, we found that the orthologous/paralogous gene pairs in all nine plant species were closely clustered, illustrating evolutionary expanded model of the *EXO* gene family. Also, the two tetraploid cotton species *G. hirsutum* and *G. barbadense* showed a significant increase (almost 2-fold) (Yang et al., 2020) in the number of identified *EXO* genes compared to their diploid progenitors (*G. arboreum* and *G. raimondii*) as well as all of the other plants included in the analysis (dicots, monocots, moss, and lycophyte). Therefore, the quantities of *GhEXO* family genes and the polyploid effect are mutually sufficient and necessary conditions. Cotton provides a powerful model system in which to study cell elongation and the effects of polyploidy.

Here, the large number of intron-less *GhEXO* genes suggests that at the early expansion stage, genes containing more introns could be lost during the passage of time (Roy and Penny, 2007; Liu et al., 2021), and these genes then formed advanced families with reduced numbers of introns (Roy and Gilbert, 2005, 2006; Tan et al., 2018). Furthermore, insertion/deletion events result in structural differences that permit the estimation of evolutionary timing (Lecharny et al., 2003; Liu et al., 2021), and the genes with fewer or no introns evolve more rapidly because introns experience weak selection pressure even though exons/introns focus more on functional roles, whether the sequence is conserved (Frugoli et al., 1998; Cai et al., 2021). The exon/intron and motif distribution patterns in most gene families are conserved, and many gene families with fewer or no introns have been identified (Li et al., 2019; Qanmber et al., 2019b; Quan et al., 2019). This is also consistent with the fact that equilibrium intron numbers in species with large population sizes will be lower, due to a host of successful intron loss mutations and few successful intron mutations (Liu et al., 2021). Therefore, we deem that the *GhEXO* gene family is a high-quality gene family, with intron loss during evolution and differentiation during the early expansion phase of evolution.

It has been reported that the gene family expands with the duplication of genes (Xu et al., 2012; Zeng et al., 2021). Previous studies have shown that cotton experienced genome duplication events during evolution, and subsequently there were increases in the sizes of many gene families. Duplicated gene pairs may exhibit functional differentiation, such as partial or complete loss of function, the gain of a new function, or conservation of the original function (Vandepoele et al., 2003; Campbell et al., 2019; Huang et al., 2021). The 39 *GhEXO* genes showed uneven distribution on the 18 chromosomes of *G. hirsutum*. Except for *GhEXO3*, *GhEXO4t*, and *GhEXO5*, all other orthologo were located on homologous chromosomes. Previously, a translocation event was identified between the second and third chromosomes of the At subgenome (Zhang et al., 2015; Yang et al., 2020), which might be the explanation that *GhEXO3* (the gene on At and Dt sub-genomes), *GhEXO4*, and *GhEXO5* are not on homologous chromosomes compared with its orthologs.

In this study, most of the *EXO* genes were found to originate from WGD or segmental duplication events, while only three genes showed evidence of having been derived from tandem duplication. However, no singletons or proximal duplication was found investigated how WGD or segmental duplication expanded many gene families, while it reported that tandem duplication generated an increased number of introns along with new genes (Iwamoto et al., 1998; Hartmann et al., 2018). Furthermore, homologous locus relationship between the At and Dt sub-genomes showed that 15 *GhEXO* genes are present in colinear blocks, indicating that these were active during evolution. Additionally, analysis of the selection pressure (positive selection, negative selection, and purifying selection) by computing the non-synonymous (Ka) to synonymous substitution (Ks) ratios (Ka/Ks) suggests that all orthologous/paralogous gene pairs had Ka > Ks values which is evidence that purifying selection pressure acted on the *GhEXO* genes which in turn conserved their function despite the expansion of the gene family. Therefore, the main method of amplification of the *GhEXO* gene family with conserved gene function is gene duplication. In summary, *GhEXO* genes are an ancient gene family that existed in the early stages of evolution and under the pressure of purifying selection. Its expansion in the genome originates from genome-wide duplication and fragment duplication and conforms to the polyploid effect.

### Diverse Expression Patterns of the *GhEXO* Genes

We investigated the expression patterns of the *GhEXO* genes in various tissues and fiber samples and found that the tested *GhEXO* genes were expressed in all tissues. However, *GhEXO12_At/Dt* and *GhEXO13_At/Dt* were advantageously expressed in 0, 5, 10, and 20 DPA fiber tissues. Similarly, *GhEXO8_At/Dt*, *GhEXO14_At/Dt*, *GhEXO16_At/Dt*, *GhEXO17_At/Dt*, *GhEXO18_At/Dt*, and *GhEXO19_At/Dt* were expressed in the roots, while all other genes had uneven expression patterns. There are almost no very similar expression patterns among family members, which means that *GhEXO* family members function in various organizations. These highly enriched fiber-related genes might be important genetic targets for fiber breeding in cotton.

It has been reported that *EXO* expression is relatively high in the tissue of high concentrations of auxin and auxin can induce the expression of *EXO* in *AtEM201* (Farrar et al., 2003); *EXO* ectopic expression was observed in the hypocotyl of etiolated seedings and in root vascular tissue of GA treatment (Farrar, 2000); BR-deficient *dwf1-6* and *CPD* antisense plants showed weaker *EXO* expression, and exogenous BL resulted in increased *EXO* transcript levels in both wild-type and *dwf1-6* plants (Coll-Garcia et al., 2004). In addition, these three phytohormones can promote plant growth and development, corresponding to the function of *EXO*. Hence, we investigated the expression patterns of *GhEXO* genes in response to exposure to three plant hormones (IAA, GA, and BL) and observed variable response patterns. For example, *GhEXO3_At/Dt*, *GhEXO4_At/Dt*, *GhEXO7_At/Dt*, *GhEXO8_At/Dt*, and *GhEXO17_At/Dt* were upregulated while *GhEXO1_At/Dt*, *GhEXO9_At/Dt*, *GhEXO10_At/Dt*, *GhEXO11_At/Dt*, *GhEXO12_At/Dt*, and *GhEXO14_At/Dt* were completely downregulated by all hormone treatments. *GhEXO3_At/Dt*, *GhEXO4_At/Dt*, *GhEXO7_At/Dt*, *GhEXO8_At/Dt*, *GhEXO15_At/Dt*, and *GhEXO17_At/Dt* showed upregulation at all time points in response to BL. These genes might be the key genes involved in the responses to the relevant hormones, and they could prove to be important genetic targets for future cotton breeding programs.

### *GhEXO7_At* Mediates BR-Regulated Plant Growth and Development

As reports, BRs was considered to promote growth primarily through cell expansion or elongation (Oh et al., 2020). BR-deficient mutants show plant dwarfing, hypocotyls and root cells are shortened to varying degrees, and the two-dimensional expansion of leaf cells is restricted (Yang et al., 2014; Oh et al., 2020). A limited number of studies have explored the function of *EXO* genes in different plant species; however, ours is the first to characterize the *EXO* gene family in cotton. A previous report indicated that inhibition of *EXO* gene expression does not cause abnormal phenotypes (Coll-Garcia et al., 2004), while *exo* knockout mutants exhibited dwarfing due to reduced cell expansion (Schroder et al., 2009). Similarly, overexpression of *EXO* genes gave plants with increased vegetative growth accompanied by increased expression of BR-up-regulated genes. Additionally, *EXO* genes show weak expression in BR-deficient mutants, while enhanced expression of *EXO* genes in BR-treated plants has been reported (Coll-Garcia et al., 2004).

In our study, MM 4-64 was used to co-locate with GhEXO7_At-GFP in *N. benthamiana* leaf cells confirmed that GhEXO7_At is a plasma membrane protein. MM 4-64 is a membrane dye that can specifically bind to the plasma membrane in a short time and emit high-intensity red fluorescence. In the early stage, it was used to study the endocytosis of yeast cells (Vida and Emr, 1995). Now, it is widely used in the medical field, such as the staining of cardiomyocytes (Chen et al., 2015). In addition, it has also been used to stain *Arabidopsis* root cells, *Brassica napus* L. seeds and tobacco cells in plant kingdom (Jelínková et al., 2010, 2015; Rigal et al., 2015; Lin et al., 2019). Ectopic expression of *GhEXO7_At* in Arabidopsis gave transgenic plants that exhibited increased vegetative growth and root growth as compared to the control Col-0 plants. Histological observations show that it has stronger cell expansion than WT and this is exactly the mechanism by which BR promotes growth. Deeper investigation revealed that BR biosynthesis genes (*DWF4* and *CPD*) showed downregulated expression, while the fatty acid elongate gene *KCS1* and the cell elongation gene *EXP5* were upregulated in *GhEXO7_At*-overexpression lines, demonstrating BR-mediated growth and development by the *GhEXO7_At* gene in *Arabidopsis*. In addition to affecting cell expansion, BRs can also affect cell division (Oh et al., 2020), but we have not observed this aspect. These results were further verified in cotton plants in which *GhEXO7_At* gene expression was decreased 70–80% by VIGS. We observed reduced plant growth and development, including reduced leaf length, fiber length, and root length, in silenced plants as compared to the control plants. However, we found that not only the transcription level of *GhEXO7_At* was suppressed in silent plants, but also other *GhEXO* genes from the same phylogenetic clade as *GhEXO7_At*. We speculate that the phenotype of silent plants is caused by the specific expression of these genes in different tissues. *GhEXO7_At* was most significantly inhibited, so we think it is the main effector gene. Taken together, we conclude that *GhEXO7_At* gene mediates BR-regulated plant growth and development in cotton.

## Conclusion

Although the function of certain *EXO* genes has previously been demonstrated in *Arabidopsis*, the function of the *EXO* gene family in cotton is unclear. In the present study, we identified 175 *EXO* genes in nine plant species, including 21 *EXO* genes in *G. arboreum*, 39 in *G. hirsutum*, 19 in *G. raimondii*, and 40 in *G. barbadense*. A phylogenetic analysis grouped the 175 EXO proteins into five major clades. Approximately 75% of the *GhEXO* genes have no introns. Conserved amino acid sequence signatures of EXO proteins from *Arabidopsis*, rice, and *G. hirsutum* showed that the EXO protein sequences are highly evolutionarily conserved. Collinearity analysis verified that segmental duplication and WGD events were the main drivers of the expansion of the cotton *EXO* gene family. Furthermore, gene expression analysis indicated that the *GhEXO* genes were without exception expressed in eight tested cotton tissues, and also evidenced that the expression of these genes can be regulated by hormone treatments, indicating that *GhEXO* genes mediate plant responses to hormonal stress. We found that GhEXO7_At is a plasma membrane-localized protein, and overexpression of *GhEXO7_At* enhanced growth in *Arabidopsis*, suggesting that it mediates the effect of BR on plant growth. Silencing *GhEXO7_At* in cotton caused a decrease in leaf length, leaf width, plant height, and fiber length. Our results will be not only helpful to understanding the evolution of cotton *EXO* genes, but will also provide important candidate genes for genetic engineering in cotton.

## Data Availability Statement

The datasets presented in this study can be found in online repositories. The names of the repository/repositories and accession number(s) can be found in the article/Supplementary Material.

## Author Contributions

SL: experimentation and writing. GC: methodology and formal analysis. ZL: writing and editing. GQ: conceptualization and writing — review, and editing. LL: software and data curation. JZ: formal analysis and review and editing. SM: methodology and data curation. ZY: supervision. FL: supervision. All authors contributed to the article and approved the submitted version.

## Conflict of Interest

The authors declare that the research was conducted in the absence of any commercial or financial relationships that could be construed as a potential conflict of interest.

## Publisher’s Note

All claims expressed in this article are solely those of the authors and do not necessarily represent those of their affiliated organizations, or those of the publisher, the editors and the reviewers. Any product that may be evaluated in this article, or claim that may be made by its manufacturer, is not guaranteed or endorsed by the publisher.

## Footnotes

1
https://www.arabidopsis.org/

2
https://phytozome-next.jgi.doe.gov/

3
https://grand.biocloud.net/

4
https://cottonfgd.org/

5
http://www.arabidopsis.org

6
http://www.ebi.ac.uk/InterProScan/

7
http://smart.emblheidelberg.de/

8
https://www.expasy.org/resources/protparam

9
https://www.evolgenius.info/evolview/

10
https://github.com/CJ-chen/TBtools

## Supplementary Material

Supplementary Figure 1 Phylogenetic analysis of EXO protein sequences from four cotton species.

Supplementary Figure 2 Chromosomal locations of predicted *GhEXO* gene family members in the *G. hirsutum* genome.

Supplementary Figure 3 Gene structure analysis of the *GhEXO* family in *G. hirsutum*. **(A)** Protein motif distribution patterns of GhEXO proteins. **(B)** Intro/exon structures of the *GhEXO* genes.

Supplementary Figure 4 The expression levels of six other *GhEXO* genes in the *GhEXO7_At* silenced plants. Student’s *t*-test: ^∗^*p* < 0.05, ^∗∗^*p* < 0.01.

Supplementary Figure 5 Multiple sequence alignments of conserved motif data from *Arabidopsis*, rice, and *G. hirsutum*.

Supplementary Figure 6 Statistical analyses of leaf length **(A)**, leaf width **(B)**, and plant height **(C)** vector control and VIGS: *GhEXO7_At* plants. Student’s *t*-test: ^∗^*p* < 0.05. **(D)** Mature fiber length in the vector control and VIGS: *GhEXO7_At* plants. Comparison of total root length **(E)** and root fresh weight **(F)** of *GhEXO7_At*-OE and WT Student’s *t*-test: ^∗^*p* < 0.05, ^∗∗^*p* < 0.01, ^∗∗∗^*p* < 0.001.

Supplementary Table 1 Oligonucleotide primers used in this study.

Supplementary Table 2 Duplication type information for the 39 *GhEXO* genes.

Supplementary Table 3 Calculated expression values and primary data from qRT-PCR assays in this study.
