# Supplementary figures and images for: Identification and Analysis of GhEXO Gene Family Indicated That GhEXO7_At Promotes Plant Growth and Development Through Brassinosteroid Signaling in Cotton (Gossypium hirsutum L.)
Source: Front Plant Sci. 2021 Sep 16;12:719889. doi: 10.3389/fpls.2021.719889 (PMC8481617; doi:10.3389/fpls.2021.719889)

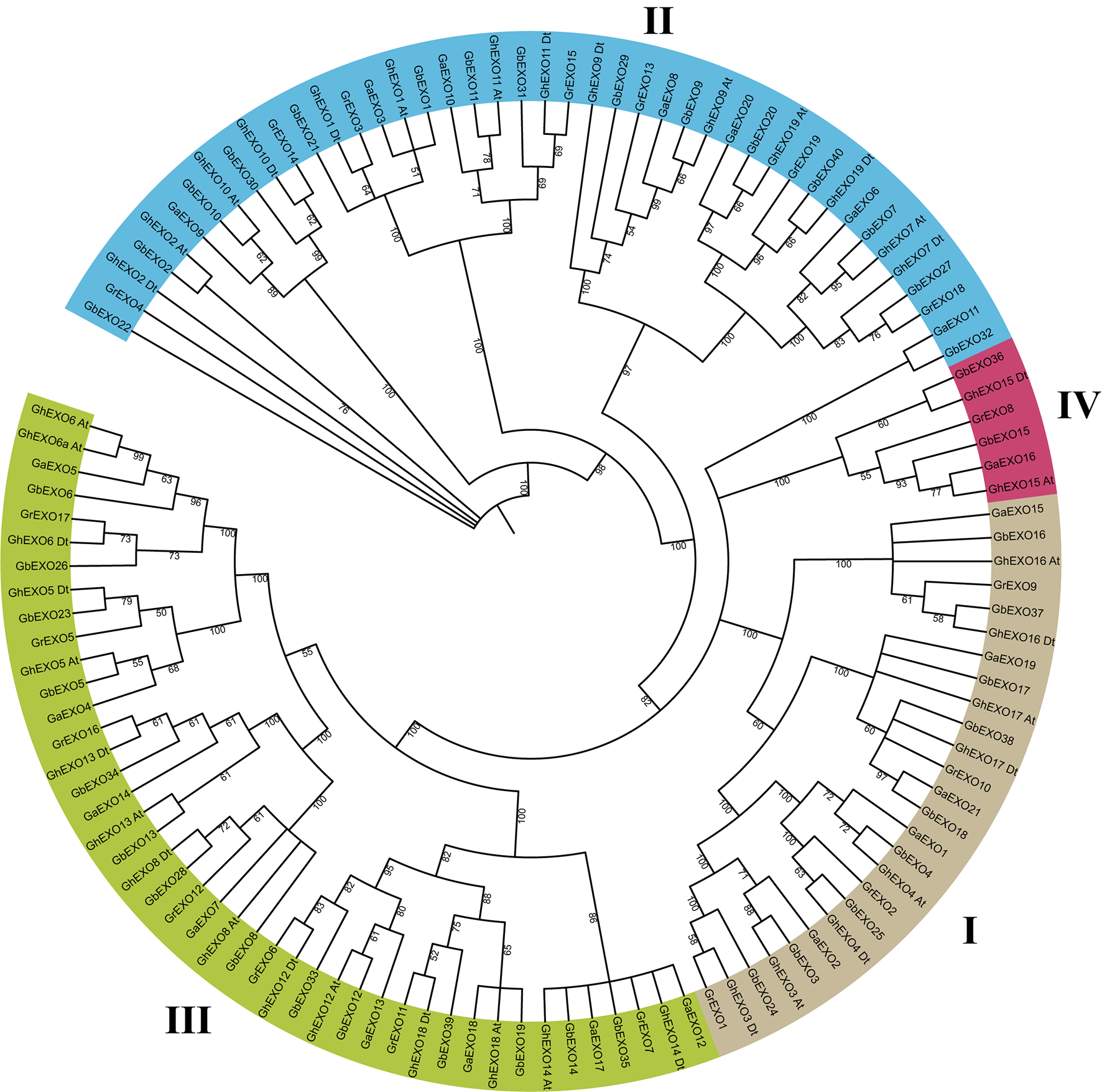

Supplement: Supplementary Figure 1 — Phylogenetic analysis of EXO protein sequences from four cotton species. [file Image_1.TIF]

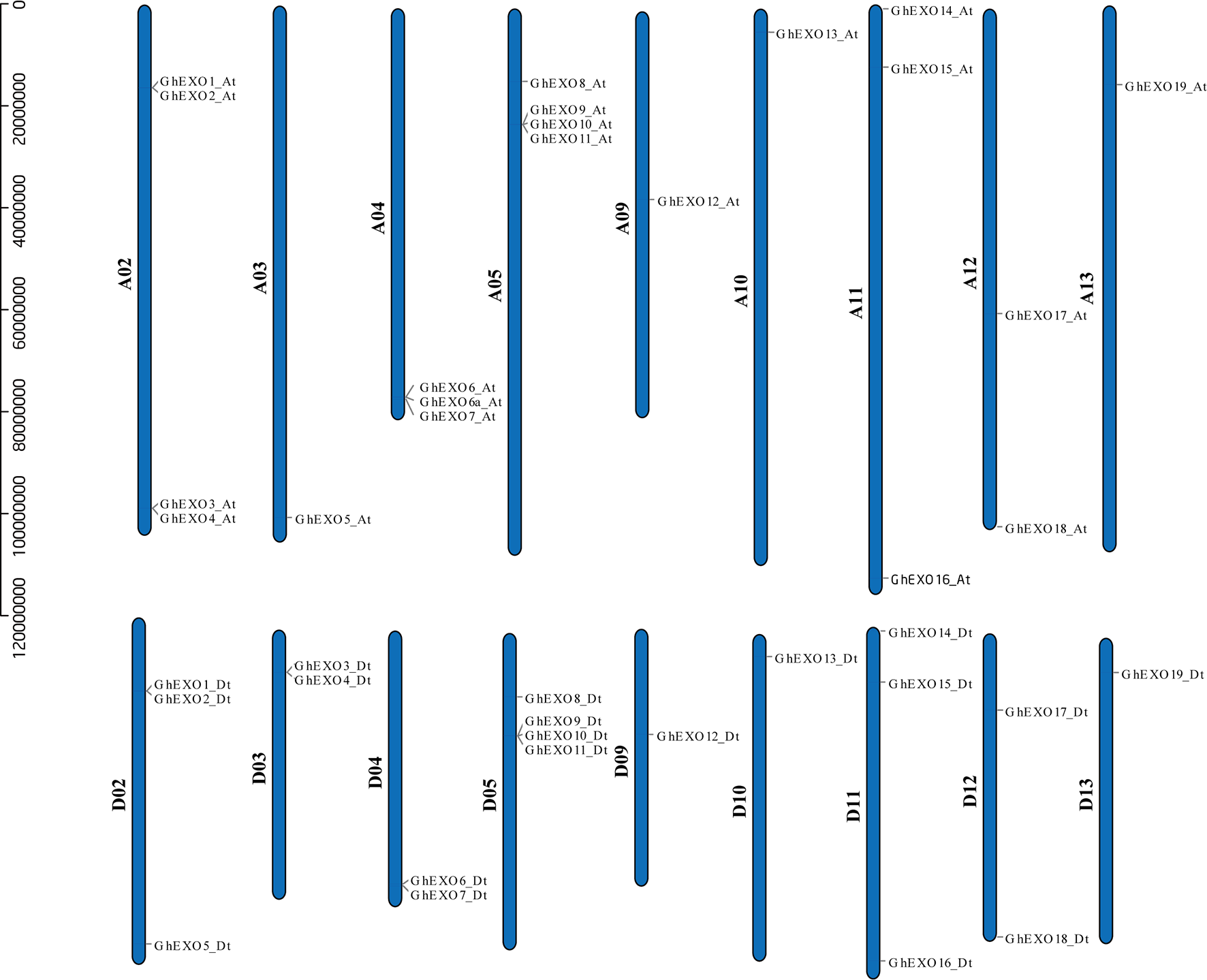

Supplement: Supplementary Figure 2 — Chromosomal locations of predicted GhEXO gene family members in the G. hirsutum genome. [file Image_2.TIF]

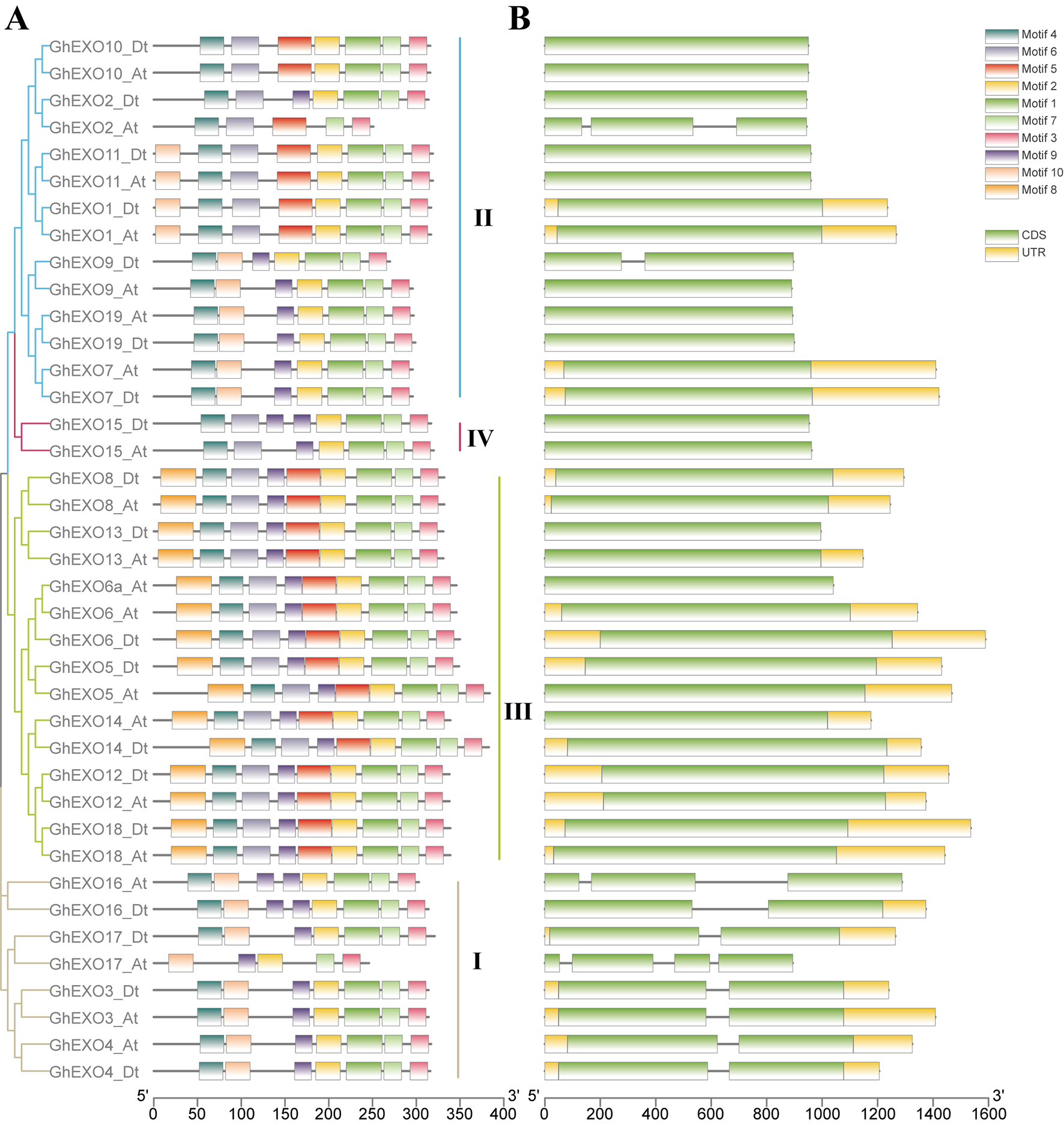

Supplement: Supplementary Figure 3 — Gene structure analysis of the GhEXO family in G. hirsutum. (A) Protein motif distribution patterns of GhEXO proteins. (B) Intro/exon structures of the GhEXO genes. [file Image_3.TIF]

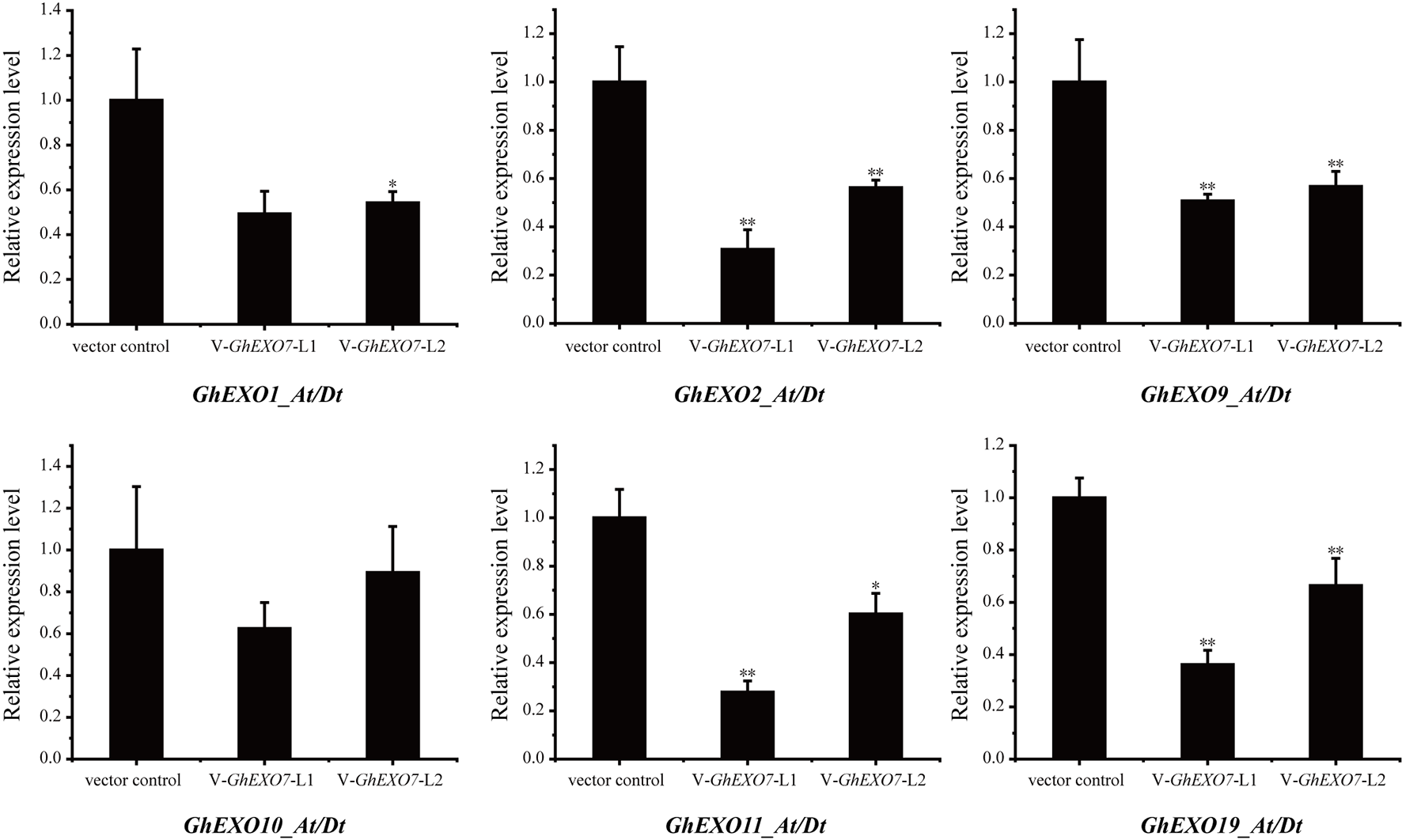

Supplement: Supplementary Figure 4 — The expression levels of six other GhEXO genes in the GhEXO7_At silenced plants. Student’s t-test: ∗p < 0.05, ∗∗p < 0.01. [file Image_4.TIF]

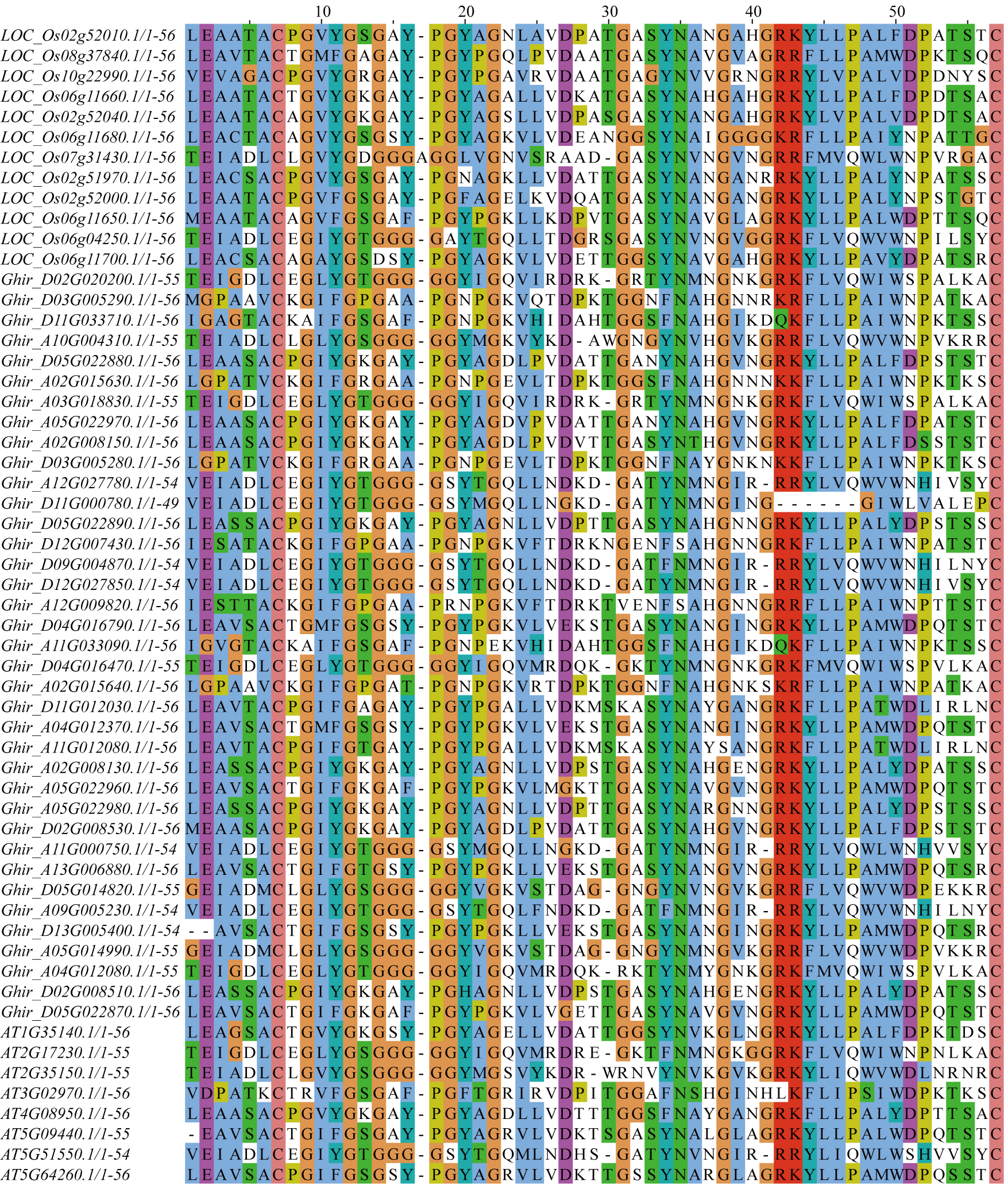

Supplement: Supplementary Figure 5 — Multiple sequence alignments of conserved motif data from Arabidopsis, rice, and G. hirsutum. [file Image_5.TIF]

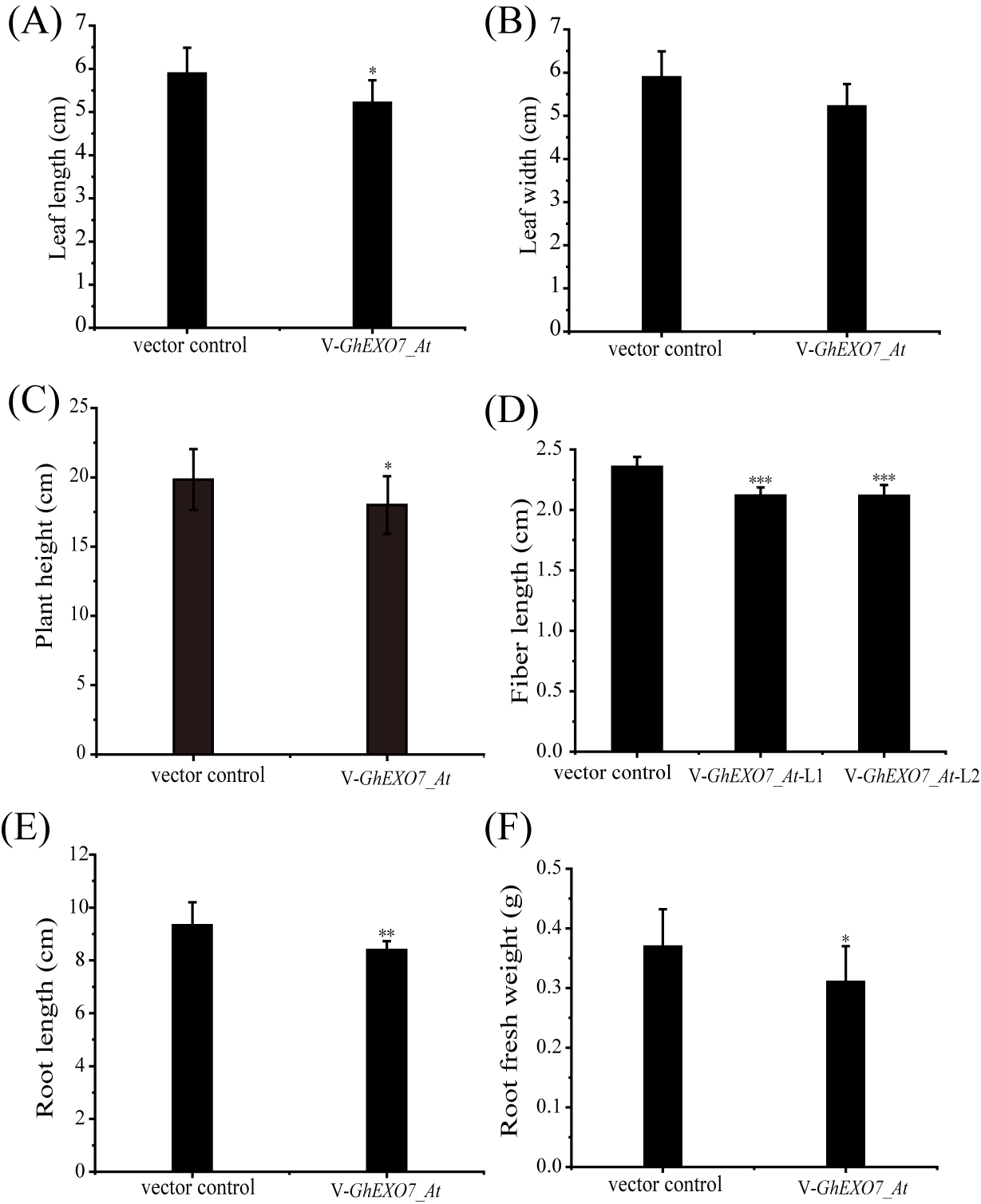

Supplement: Supplementary Figure 6 — Statistical analyses of leaf length (A), leaf width (B), and plant height (C) vector control and VIGS: GhEXO7_At plants. Student’s t-test: ∗p < 0.05. (D) Mature fiber length in the vector control and VIGS: GhEXO7_At plants. Comparison of total root length (E) and root fresh weight (F) of GhEXO7_At-OE and WT Student’s t-test: ∗p < 0.05, ∗∗p < 0.01, ∗∗∗p < 0.001. [file Image_6.TIF]
