# Supplementary material for: Identification and Analysis of GhEXO Gene Family Indicated That GhEXO7_At Promotes Plant Growth and Development Through Brassinosteroid Signaling in Cotton (Gossypium hirsutum L.)
Source: Front Plant Sci. 2021 Sep 16;12:719889. doi: 10.3389/fpls.2021.719889 (PMC8481617; doi:10.3389/fpls.2021.719889)
Supplement: Supplementary Table 1 — Oligonucleotide primers used in this study. [file Table_1.docx]

| Genes | Forward (from 5’ to 3’) | Reverse (from 5’ to 3’) |
| --- | --- | --- |
| *GhEXO1* | GGAGCAGCCTCAGCTCTTGAAA | CCTCTCGGAAGGCCTGAACTTG |
| *GhEXO2* | AGCCTTCTCAACGTGCCATTGT | GTAGCAACCGAGGGTTGAGGTG |
| *GhEXO3* | CGGCCATGGTCACACTTACGAT | GTCTTTGCCTGGGACGGACAAT |
| *GhEXO4* | TCAACCCGCCTCCTTGTTCTTG | ACCAACGGGTGACTTGAGGTTG |
| *GhEXO5* | CGGTCCTTTCTTCTTCGCCGAT | CAGAAAGGCGCGTGAGGTGATA |
| *GhEXO6* | TTCCGACTGGTGGAAAACCGTC | GGGAATAACCCGTGTCGGAGTG |
| *GhEXO7* | CCATCCAACGCTCCATAATCGT | CCGAGCGGGTAATTTTCAAGAAGG |
| *GhEXO8* | TCACCGATAGCCTTTCCCTTTCG | GCACCAAATAGAGACCACTGCG |
| *GhEXO9* | CTCTCTCAGCAACCCCAAGACG | TTTGCTTCCCTACAACGACGCT |
| *GhEXO10* | AAGGGTTCTGCTCAAGCAGGTG | GGCACTGTGTCTCGGAGTTACC |
| *GhEXO11* | TGCCCAATGGTGGAAAACCACT | CGGCTCATGCAGAACCCTTCAA |
| *GhEXO12* | CAACGGTGACGTAGGAGTGGAC | TCACCAACGGGTTAGTTGCCAG |
| *GhEXO13* | GGAACTCAATGTCCAGGGGTCG | GGTTGGTGCGGTTGGATCTTCT |
| *GhEXO14* | ACGACCGTTTTTACTCCCACCG | CTTGGCCGCAAAAATCCTCGAC |
| *GhEXO15* | GCCTTATGCTCTGCCGGCTTAT | CCCTGCAAGTAGGGTGGCTATG |
| *GhEXO16* | GGGATCGTCGGTAACATCACACC | AGCTGCCTTGTCCATGTTTGGT |
| *GhEXO17* | GCTGGAAAATGTGCTGACCACG | ATGCGCTTGCCAATGCAACTAC |
| *GhEXO18* | CTCTTGCTCACCTCCGACGATG | CCACGATTGAGGGGAACGTGAA |
| *GhEXO19* | GCGTTATCCTCACGGCCAAAGA | GCCCCTGGAAATACCCGTTGTT |
| *CPD (AT5G05690)* | CGTTTAGAGCGGTTCATTTAG | GTTTCTGCGTTCTTGTAGTTG |
| *DWF4 (AT3G50660)* | TCTTCAGTCACGAGCAACG | TCTCATGTCCGGCAAATAAC |
| *KCS1(AT1G01120)* | TTGCAACGTGACCACCATTC | GAGTTGAACCGCCTGGTTAGA |
| *EXP5 (AT3G29030)* | TGATGCTTCCGGCACTATGG | TGGTCGAATAACGCTGTGCT |
| *Actin2 (AT3G18780)* | TGTGCCAATCTACGAGGGTTT | TTTCCCGCTCTGCTGTTGT |
| *GhHiston3 (Gh_D03G0370)* | TCAAGACTGATTTGCGTTTCCA | GCGCAAAGGTTGGTGTCTTC |
| *VIGS-GhEXO7_At* | GGACGTGGTCCTGTTCG | CCACATCGCCGGCAACAAAT |

**Table. S1 Oligonucleotide primers used in this study.**
