# Supplementary material for: Identification and Analysis of GhEXO Gene Family Indicated That GhEXO7_At Promotes Plant Growth and Development Through Brassinosteroid Signaling in Cotton (Gossypium hirsutum L.)
Source: Front Plant Sci. 2021 Sep 16;12:719889. doi: 10.3389/fpls.2021.719889 (PMC8481617; doi:10.3389/fpls.2021.719889)
Supplement: Supplementary Table 2 — Duplication type information for the 39 GhEXO genes. [file Table_2.docx]

Table S2 Duplication type information of GhEXO genes.

| Gene | Gene type | chromosome | Gene | Gene type | chromosome |
| --- | --- | --- | --- | --- | --- |
| GhEXO1_At | 4 | A02 | GhEXO1_Dt | 4 | D02 |
| GhEXO2_At | 4 | A02 | GhEXO2_Dt | 4 | D02 |
| GhEXO3_At | 4 | A02 | GhEXO3_Dt | 4 | D03 |
| GhEXO4_At | 3 | A02 | GhEXO4_Dt | 3 | D03 |
| GhEXO5_At | 4 | A03 | GhEXO4_Dt | 4 | D02 |
| GhEXO6_At | 4 | A04 | GhEXO6_Dt | 4 | D04 |
| GhEXO7_At | 4 | A04 | GhEXO7_Dt | 4 | D04 |
| GhEXO8_At | 4 | A05 | GhEXO8_Dt | 4 | D05 |
| GhEXO9_At | 4 | A05 | GhEXO9_Dt | 4 | D05 |
| GhEXO10_At | 4 | A05 | GhEXO10_Dt | 4 | D05 |
| GhEXO11_At | 3 | A05 | GhEXO11_Dt | 4 | D05 |
| GhEXO12_At | 4 | A09 | GhEXO12_Dt | 4 | D09 |
| GhEXO13_At | 4 | A10 | GhEXO13_Dt | 4 | D10 |
| GhEXO14_At | 4 | A11 | GhEXO14_Dt | 4 | D11 |
| GhEXO15_At | 4 | A11 | GhEXO15_Dt | 4 | D11 |
| GhEXO16_At | 4 | A11 | GhEXO16_Dt | 4 | D11 |
| GhEXO17_At | 1 | A12 | GhEXO17_Dt | 1 | D12 |
| GhEXO18_At | 4 | A12 | GhEXO18_Dt | 4 | D12 |
| GhEXO19_At | 4 | A13 | GhEXO19_Dt | 4 | D13 |

Note: 0, 1, 2, 3, 4 stands for singleton, dispersed, proximal, tandem, WGD or segmental.
